# From immune suppression to immunotherapy sensitization: the dual roles of circRNAs in cancer progression

**DOI:** 10.3389/fimmu.2025.1723383

**Published:** 2025-12-08

**Authors:** Quan Dai, Xiaoli Yuan, Hang Dong, Haiyi Xue

**Affiliations:** 1 Department of Ultrasound, Sichuan Clinical Research Center for Cancer, Sichuan Cancer Hospital & Institute, Sichuan Cancer Center, University of Electronic Science and Technology of China, Chengdu, China; 2 Department of General Internal Medicine, Sichuan Clinical Research Center for Cancer, Sichuan Cancer Hospital and Institute, Affiliated Cancer Hospital of University of Electronic Science and Technology of China, Chengdu, China; 3 Department of Intensive Care Unit, Sichuan Clinical Research Center for Cancer, Sichuan Cancer Hospital and Institute, Affiliated Cancer Hospital of University of Electronic Science and Technology of China, Chengdu, China

**Keywords:** circular RNAs (circRNAs), tumor immune microenvironment, PD-L1 regulation and immune escape, immunotherapy sensitization, biomarkers and RNA therapeutics

## Abstract

Circular RNAs (circRNAs) have recently emerged as critical regulators of tumor–immune interactions. Owing to their covalently closed structure, remarkable stability, and tissue-specific expression, circRNAs not only serve as molecular sponges and protein regulators but also play multifaceted roles in shaping the tumor immune microenvironment. Accumulating evidence indicates that circRNAs drive immune suppression by stabilizing PD-L1 through post-translational modifications and RNA-binding protein interactions, transmitting suppressive signals via exosomes to T cells and myeloid-derived suppressor cells, reprogramming glucose and lipid metabolism to deprive effector lymphocytes, and reinforcing cancer stemness and therapy resistance. In striking contrast, a subset of circRNAs has been shown to sensitize tumors to immunotherapy by activating innate immune pathways such as RIG-I/MAVS and STING, inducing immunogenic cell death, and overcoming resistance to endocrine therapy or ferroptosis inducers, thereby enhancing the efficacy of immune checkpoint blockade. Beyond their mechanistic functions, circRNAs also hold promise as stable and accessible biomarkers for prognosis, patient stratification, and therapeutic monitoring, particularly when enriched in circulating exosomes. Advances in antisense oligonucleotides, RNA interference, and nanomedicine provide new opportunities to therapeutically target oncogenic circRNAs or deliver engineered pro-immunogenic circRNAs. While significant challenges remain in detection accuracy, functional annotation, delivery specificity, and clinical validation, circRNAs represent a new frontier in immuno-oncology. Harnessing their dual roles may unlock innovative biomarker platforms and next-generation RNA-based therapeutics, ultimately improving the efficacy of cancer immunotherapy.

## 1 Introduction

Cancer remains one of the leading causes of morbidity and mortality worldwide, accounting for nearly 20 million new cases and 10 million deaths annually according to the latest GLOBOCAN statistics (1–4). Despite substantial advances in surgery, radiotherapy, chemotherapy, and targeted therapies, the prognosis for many advanced malignancies remains poor, with 5-year survival rates below 20% for certain tumor types such as lung, pancreatic, and hepatocellular carcinoma (5–9). The advent of immunotherapy, particularly immune checkpoint inhibitors (ICIs) targeting PD-1/PD-L1 and CTLA-4 pathways, has revolutionized treatment paradigms and improved outcomes in subsets of patients (10). However, only a fraction of patients achieve durable responses, and primary or acquired resistance to ICIs continues to limit their broad clinical benefit. This pressing clinical challenge underscores the urgent need to identify novel molecular regulators of immune evasion and therapeutic sensitivity—among which circular RNAs (circRNAs) are emerging as promising candidates (11–13). CircRNAs have recently emerged as a unique class of noncoding RNAs with important regulatory potential in cancer biology. Unlike linear RNAs, circRNAs are generated by back-splicing and form covalently closed loop structures that confer remarkable resistance to exonuclease digestion. This structural feature endows circRNAs with exceptional stability, evolutionary conservation, and tissue- or disease-specific expression profiles, making them attractive candidates for mechanistic studies as well as diagnostic and therapeutic applications. Beyond their canonical function as microRNA sponges, circRNAs have been reported to regulate gene transcription, modulate protein stability, and even encode small peptides, highlighting their functional versatility.

The tumor immune microenvironment (TIME) plays a decisive role in shaping tumor progression and determining therapeutic responses. It is composed of a dynamic network of tumor cells, immune and stromal cells, extracellular matrix, and soluble mediators that collectively orchestrate the balance between effective antitumor immunity and immune tolerance (14–16). Within this ecosystem, immune escape arises through multiple, often overlapping mechanisms. One central strategy is the upregulation of inhibitory immune checkpoints such as PD-1/PD-L1 and CTLA-4, which directly impair T cell activation and cytotoxicity. In parallel, the recruitment and functional reprogramming of immunosuppressive cell subsets—including myeloid-derived suppressor cells (MDSCs), regulatory T cells (Tregs), and tumor-associated macrophages (TAMs)—create an environment that actively suppresses effector T cell responses and promotes tumor survival. Metabolic reprogramming further exacerbates this imbalance: cancer cells consume excessive glucose, amino acids, and lipids to sustain rapid proliferation, thereby depriving CD8^+^ T cells and natural killer (NK) cells of essential nutrients, while generating immunosuppressive metabolites such as lactate, adenosine, and kynurenine (17–19). These metabolic by-products impair effector cell function and promote the differentiation of suppressive cell populations. Additionally, stromal components and cytokines, including TGF-β, IL-10, and VEGF, remodel the extracellular matrix and vasculature to physically and functionally exclude immune cells from tumor nests, reinforcing immune evasion (20–22). Collectively, these processes enable tumor cells to escape immune surveillance and drive resistance to immunotherapies, particularly ICIs (23–25). Although ICIs have transformed treatment paradigms and provided durable clinical benefit in subsets of patients, the majority either fail to respond or develop acquired resistance. Understanding the cellular, molecular, and metabolic mechanisms underpinning immune escape is therefore critical to identifying new targets and strategies to broaden the efficacy of immunotherapy.

Accumulating evidence now demonstrates that circRNAs are deeply integrated into the immunological processes that govern tumor progression and therapeutic outcomes. On one hand, numerous oncogenic circRNAs reinforce immune suppression through diverse mechanisms. They stabilize or upregulate PD-L1 via post-transcriptional regulation and post-translational modifications, thereby directly inhibiting T cell–mediated cytotoxicity (26). Others promote T cell exhaustion by transmitting immunosuppressive signals through tumor-derived exosomes or by sustaining inhibitory signaling cascades within the TME (27). CircRNAs also influence the polarization of immunosuppressive cells, including regulatory T cells, myeloid-derived suppressor cells, and tumor-associated macrophages, amplifying local immune tolerance. In addition, by rewiring tumor metabolism—such as enhancing glycolysis or fatty acid uptake—circRNAs indirectly deprive effector lymphocytes of nutrients while fueling tumor growth, further tipping the balance toward immune escape. On the other hand, an emerging subset of circRNAs exerts tumor-suppressive and immunostimulatory functions, acting as sensitizers of immunotherapy. These circRNAs can activate innate immune signaling pathways such as RIG-I/MAVS and STING, leading to interferon production and enhanced antigen presentation (28). Others promote immunogenic cell death, exposing tumor antigens and danger-associated molecular patterns that facilitate dendritic cell activation and T cell priming. Furthermore, certain circRNAs restore therapeutic sensitivity by reversing drug resistance or metabolic adaptations, thereby augmenting the efficacy of immune checkpoint blockade and other immunotherapies. This duality highlights circRNAs as context-dependent regulators of cancer immunity, positioning them as both drivers of immune evasion and potential allies in sensitizing tumors to immunotherapy.

The purpose of this review is threefold: (i) to summarize the molecular mechanisms by which circRNAs promote tumor immune suppression, (ii) to highlight recent findings that demonstrate their ability to enhance immunotherapy responsiveness, and (iii) to discuss the translational potential of circRNAs as biomarkers and therapeutic targets in the context of cancer immunology. By integrating these perspectives, we aim to provide a comprehensive overview of the multifaceted roles of circRNAs in cancer immunity and to outline future directions for research and clinical application.

## 2 circRNAs as drivers of tumor immune suppression

A growing body of evidence indicates that circRNAs act as critical regulators of immune suppression within the tumor microenvironment. These molecules interfere with antitumor immunity through multiple layers of regulation, including immune checkpoint modulation, exosome-mediated intercellular communication, metabolic reprogramming, and the maintenance of stem-like and drug-resistant phenotypes (29–32). **Table 1** systematically summarizes the major mechanistic classes through which circRNAs drive tumor immune evasion across different cancer types.

**Table 1:** Mechanisms and representative molecules of circRNAs in tumor immunosuppression.

Mechanism	circRNA	Specific mechanism	Cancer types
Regulating immune checkpoints	hsa_circ_0136666	Promoting PD-L1 phosphorylation and stability through miR-375/PRKDC axis	Gastric cancer (33)
	circIGF2BP3	M ^6^ A modified circIGF2BP3 enhances PD-L1 deubiquitination and stability by recruiting OTUB1, thereby inhibiting CD8 ^+^ T cell response and resistance to PD-1 blockade	Non-small cell lung cancer (34)
	circRHBDD1	Stabilize PD-L1 mRNA through IGF2BP2	Gastric cancer (35)
	circQSOX1	Promoting glucose metabolism and Treg infiltration through miR-326/330-5p/PGAM1 axis, leading to anti-CTLA-4 resistance	Colorectal cancer (36)
Regulating intercellular signaling pathways	exosomal circUSP7	Induction of CD8 ^+^ T cell depletion through miR-934/SHP2 axis	Non-small cell lung cancer (37)
	exosomal circ_0001947	Promote CD8 ^+^ T cell depletion and resist PD-1 therapy	Gastric cancer (27)
	exosomal circRNA_0013936	Enhancing the immunosuppressive function of PMN MDSCs through FATP2/RIPK3 axis	Bladder cancer (38)
	exosomal circ-0100519	Induction of polarization of M2 macrophages via USP7/NRF2 axis	Breast cancer (39)
Regulate metabolic reprogramming	circRUNX1	Enhance glycolysis through miR-145/HK2 axis and inhibit CD8 ^+^ T cells through lactate accumulation	Non-small cell lung cancer (31)
	circZNF609	Promoting fatty acid uptake through IGF2BP2/CD36 axis and reducing energy supply to effector T cells	Bladder cancer (40)
	circ_0008287	Reshaping TME through miR-548c-3p/CLIC1 axis promotes immune escape	Gastric cancer (41)
Promote tumor stemness and drug resistance	circFAT1	Activate the STAT3 signaling pathway to enhance stemness and immune tolerance	Multiple types of cancer (42)
	circCFL1	Stabilize c-Myc through deubiquitination, promote transcription and immune escape of mutant TP53	Triple negative breast cancer (43)
	circPHLPP2	By combining with ILF3, upregulate IL36 γ and promote anti-PD-1 resistance	Colorectal cancer (44)

### 2.1 Regulation of immune checkpoints

**Figure 1:**
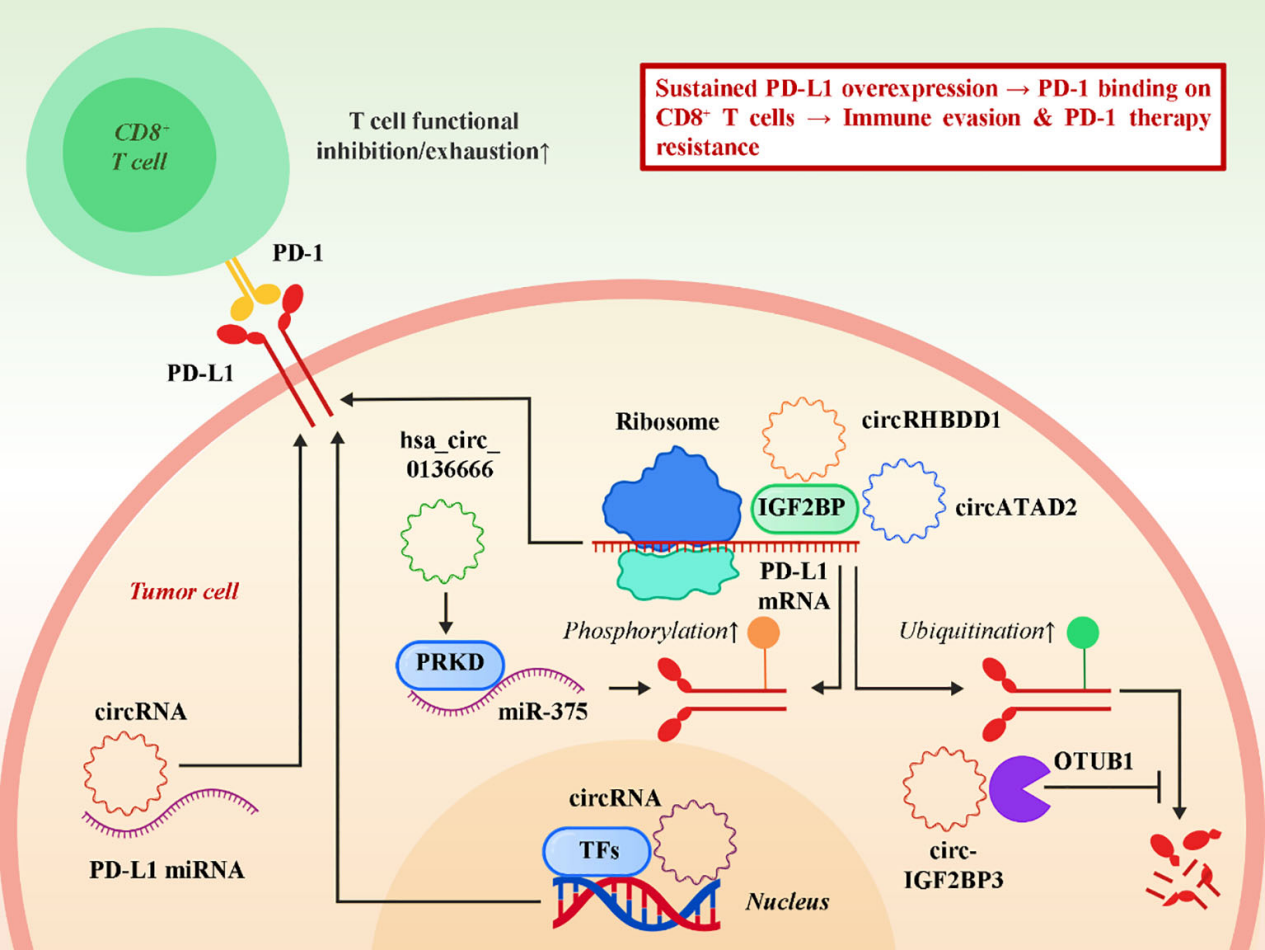
circRNAs regulate PD-L1 expression and stability to promote tumor immune escape.

One of the most direct and well-characterized mechanisms by which circRNAs promote immune evasion is through the regulation of programmed death-ligand 1 (PD-L1), a critical immune checkpoint molecule that enables tumor cells to inhibit T cell–mediated cytotoxicity (45). In many cancers, circRNAs act as upstream modulators of PD-L1 expression and stability at multiple regulatory levels, including transcriptional, post-transcriptional, and post-translational control. Several circRNAs have been shown to enhance PD-L1 expression by functioning as competing endogenous RNAs (ceRNAs) that sequester tumor-suppressive microRNAs, thereby preventing them from repressing PD-L1 (46, 47). Others interact directly or indirectly with RNA-binding proteins and deubiquitinating enzymes to extend PD-L1 protein half-life, protecting it from proteasomal degradation. For instance, hsa_circ_0136666 in gastric cancer facilitates PD-L1 phosphorylation via the miR-375/PRKDC axis, which not only stabilizes PD-L1 but also augments its membrane localization and inhibitory activity toward T cells, ultimately promoting tumor immune escape (33, 47). Similarly, the m^6A-modified circIGF2BP3 in non–small cell lung cancer (NSCLC) enhances PD-L1 deubiquitination and stabilization through the recruitment of OTUB1, dampening CD8^+^ T cell responses and contributing to resistance against PD-1 blockade (34, 46). These findings underscore how circRNAs integrate with post-translational modifications to preserve PD-L1 activity. In addition, circRNAs such as circRHBDD1 and circATAD2 exert their effects at the mRNA level by binding to members of the IGF2BP protein family (35, 46). By stabilizing PD-L1 transcripts and protecting them from degradation, these circRNAs establish a post-transcriptional regulatory hub that appears conserved across multiple tumor types. This IGF2BP-dependent mechanism suggests that circRNAs may converge on common pathways to reinforce PD-L1 expression irrespective of tumor origin. Beyond these examples, other circRNAs have been reported to regulate upstream transcription factors or signaling cascades, indirectly driving PD-L1 upregulation in diverse malignancies (48). Beyond PD-L1, emerging evidence suggests that circRNAs can also modulate other immune checkpoints, thereby broadening their impact on tumor immune escape. Notably, the N6-methyladenosine (m^6A)-modified circQSOX1 was recently reported to promote colorectal cancer resistance to anti-CTLA-4 therapy (36). In this pathway, circQSOX1 is stabilized by METTL3-dependent m^6A modification and protected by the m^6A reader IGF2BP2, enabling circQSOX1 to sponge miR-326 and miR-330-5p. This releases their inhibition of PGAM1, a key glycolytic enzyme, thereby enhancing glycolytic flux and fostering an immunosuppressive tumor microenvironment enriched in regulatory T (Treg) cells. The accumulation of intratumoral Tregs not only dampens cytotoxic immune responses but also blunts the therapeutic efficacy of CTLA-4 blockade. Importantly, genetic silencing of circQSOX1 restores sensitivity to anti-CTLA-4 therapy, underscoring the potential of targeting circRNA-mediated metabolic reprogramming to overcome immune checkpoint resistance. Together, these findings expand the scope of circRNA-mediated immune regulation beyond the PD-1/PD-L1 axis and highlight the need for further exploration into how circRNAs orchestrate multi-faceted control of immune checkpoint pathways. **Figure 1** illustrates a comprehensive model of how circRNAs orchestrate the upregulation of PD-L1 and other immune checkpoints to drive immune evasion.

### 2.2 Exosomal circRNAs as immunosuppressive messengers

**Figure 2:**
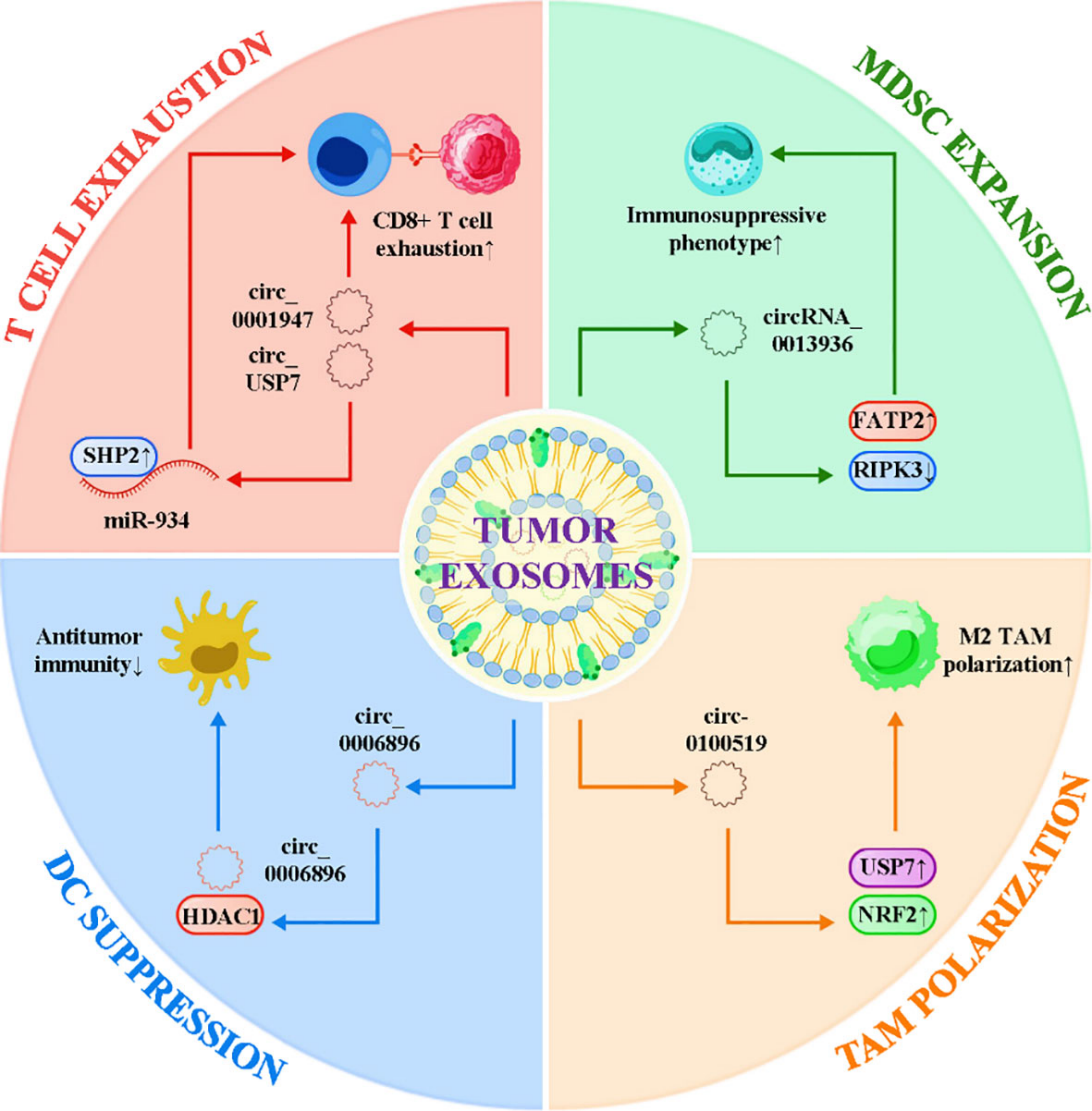
Exosomal circRNAs act as immunosuppressive messengers to reprogram immune cells and promote immune evasion.

Exosomes, a major subtype of extracellular vesicles (EVs), represent an essential vehicle through which circRNAs are transported and disseminated within the tumor microenvironment. These nanosized vesicles (30–150 nm) originate from endosomal compartments and are secreted in large quantities by tumor cells. They encapsulate circRNAs together with proteins, DNA fragments, and other noncoding RNAs, enabling long-range intercellular communication (49–52). Owing to their stability and protection from nuclease degradation, exosomal circRNAs can persist in circulation and profoundly influence immune cell behavior, thereby shaping systemic as well as local immunosuppressive niches. Tumor-derived exosomal circRNAs reprogram the activity of immune cells in favor of tolerance and immune escape. In NSCLC, exosomal circUSP7 was shown to impair CD8^+^ T cell function by regulating the miR-934/SHP2 axis, thereby inducing T cell exhaustion and driving resistance to anti-PD-1 therapy (37). In bladder cancer, exosomal circRNA_0013936 enhances the immunosuppressive phenotype of polymorphonuclear myeloid-derived suppressor cells (PMN-MDSCs) through metabolic reprogramming, upregulating FATP2 to boost lipid uptake while suppressing necroptosis via RIPK3 downregulation (38). In gastric cancer, small extracellular vesicles enriched in circ_0001947 promote CD8^+^ T cell exhaustion and confer resistance to PD-1 blockade, highlighting the capacity of exosomal circRNAs to disseminate resistance traits within the TME (27). Beyond T cells and MDSCs, exosomal circRNAs also modulate the polarization of macrophages and dendritic cell function. For instance, in breast cancer, exosomal circ-0100519 was reported to induce M2 macrophage polarization via the USP7/NRF2 axis, thereby skewing the immune microenvironment toward a suppressive state (39). Similarly, in acute myeloid leukemia (AML), exosomal circ_0006896 interacts with HDAC1 to limit antitumor immunity, providing evidence that circRNA-containing EVs can also reprogram myeloid cell differentiation and epigenetic states (53). These findings expand the immunosuppressive spectrum of exosomal circRNAs beyond T cell exhaustion, underscoring their systemic influence across multiple immune cell lineages. Importantly, exosomal circRNAs have been implicated in mediating therapeutic resistance by exporting resistance traits from tumor cells to immune cells. Their stability in biofluids makes them attractive as noninvasive biomarkers for monitoring tumor–immune interactions and predicting patient responsiveness to immunotherapy. Indeed, several studies have correlated high levels of specific exosomal circRNAs with poor clinical outcomes or lack of response to immune checkpoint inhibitors (54). This highlights the translational potential of exosomal circRNAs not only as mechanistic drivers of immune suppression but also as accessible biomarkers for liquid biopsy applications. **Figure 2** illustrates the role of exosomal circRNAs as immunosuppressive messengers in the tumor microenvironment. Collectively, these examples underscore the role of exosomal circRNAs as potent “messengers” of immune suppression and therapeutic resistance. By transferring regulatory cargo between tumor and immune cells, exosomal circRNAs extend the immunomodulatory capacity of cancer cells beyond the tumor mass itself, orchestrating systemic tolerance. Targeting exosomal circRNA biogenesis, release, or uptake therefore represents a promising therapeutic avenue to disrupt tumor–immune crosstalk and restore effective antitumor immunity.

### 2.3 CircRNAs coordinate tumor metabolism and immune microenvironment remodeling

**Figure 3:**
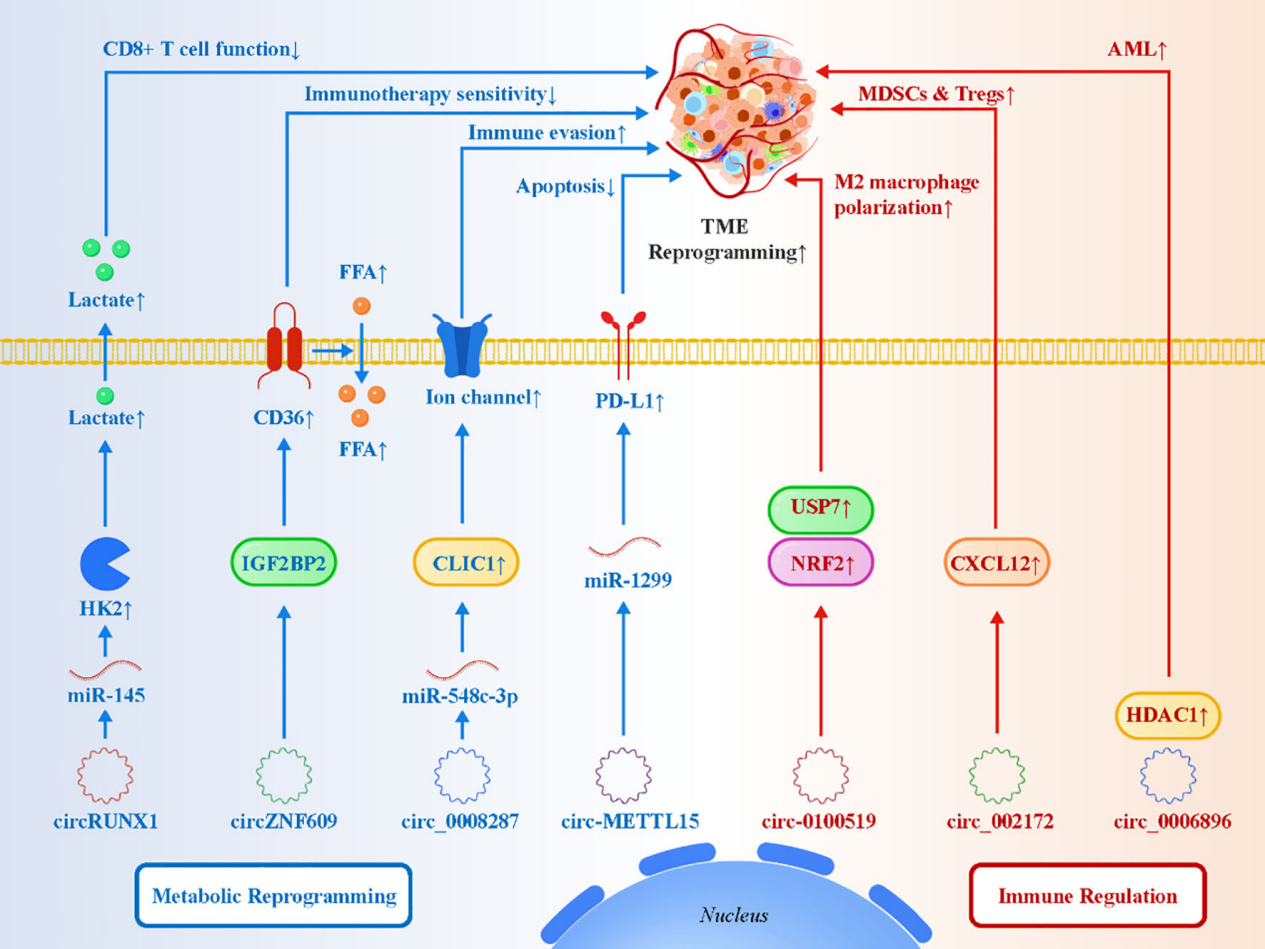
circRNAs reshape the tumor microenvironment (TME) through metabolic reprogramming and immune cell modulation.

Beyond their roles in immune checkpoint regulation, circRNAs remodel the tumor microenvironment (TME) by coordinating metabolic reprogramming and shaping the differentiation and function of immune cells. Metabolic rewiring—including enhanced glycolysis, altered fatty acid metabolism, and lactate accumulation—is a defining feature of the TME, and circRNAs are increasingly recognized as upstream regulators of these processes (55–60). By modulating key metabolic enzymes, transporters, and metabolic signaling cascades, circRNAs simultaneously support tumor biomass accumulation and dampen antitumor immunity. One representative example is circRUNX1, which enhances glycolytic flux in NSCLC via the miR-145/HK2 axis, leading to increased glucose consumption and lactate production (31). Accumulated lactate not only fuels tumor proliferation but also activates lactate–GPR81 signaling in immune cells, suppressing CD8^+^ T-cell cytotoxicity and facilitating their transition toward an exhausted phenotype. Similarly, circZNF609 stabilizes the IGF2BP2/CD36 complex in bladder cancer to promote fatty acid uptake, thereby activating PPAR signaling and supporting lipid-dependent tumor growth while impairing T-cell metabolic fitness and reducing immunotherapy responsiveness (40). CircRNAs also modulate the immune composition of the TME through direct regulation of myeloid and lymphoid cells. Exosomal circ-0100519 promotes M2 macrophage polarization via the USP7/NRF2 pathway in breast cancer, activating downstream antioxidant and metabolic genes that reinforce an immunosuppressive macrophage phenotype (39). Likewise, circ_002172 upregulates the CXCL12–CXCR4 chemokine axis in breast and colorectal cancers, driving the recruitment of regulatory T cells (Tregs) and myeloid-derived suppressor cells (MDSCs) into the tumor niche (61). These effects synergize with tumor metabolic rewiring: nutrient competition and lactate-rich conditions further stabilize M2 macrophages and suppress effector T-cell infiltration. Additional studies demonstrate that circRNAs can integrate metabolic and immunological regulation. In gastric cancer, circ_0008287 activates the miR-548c-3p/CLIC1 axis to regulate chloride channel signaling, promoting ROS modulation and immune evasion (41). In lung cancer, circ-METTL15 enhances PD-L1 expression through the miR-1299/PD-L1 pathway and stimulates PI3K–Akt signaling, simultaneously promoting tumor proliferation and reinforcing immune tolerance (62). These findings highlight the ability of circRNAs to coordinate metabolic pathways, immune checkpoint regulation, and immune cell recruitment in an integrated manner. **Figure 3** highlights the dual role of circRNAs in coordinating tumor metabolism and immune suppression within the TME. Collectively, these examples demonstrate that circRNAs act as versatile regulators of the TME by linking metabolic rewiring to immunological remodeling. By simultaneously fueling tumor growth and suppressing antitumor immunity, circRNAs help establish a metabolic–immune feedback loop that sustains immune escape. Targeting circRNA-mediated metabolic and immunological crosstalk thus represents a promising strategy to recondition the TME and improve the efficacy of immunotherapy.

### 2.4 CircRNAs drive cancer stemness, therapeutic resistance, and immune escape

**Figure 4:**
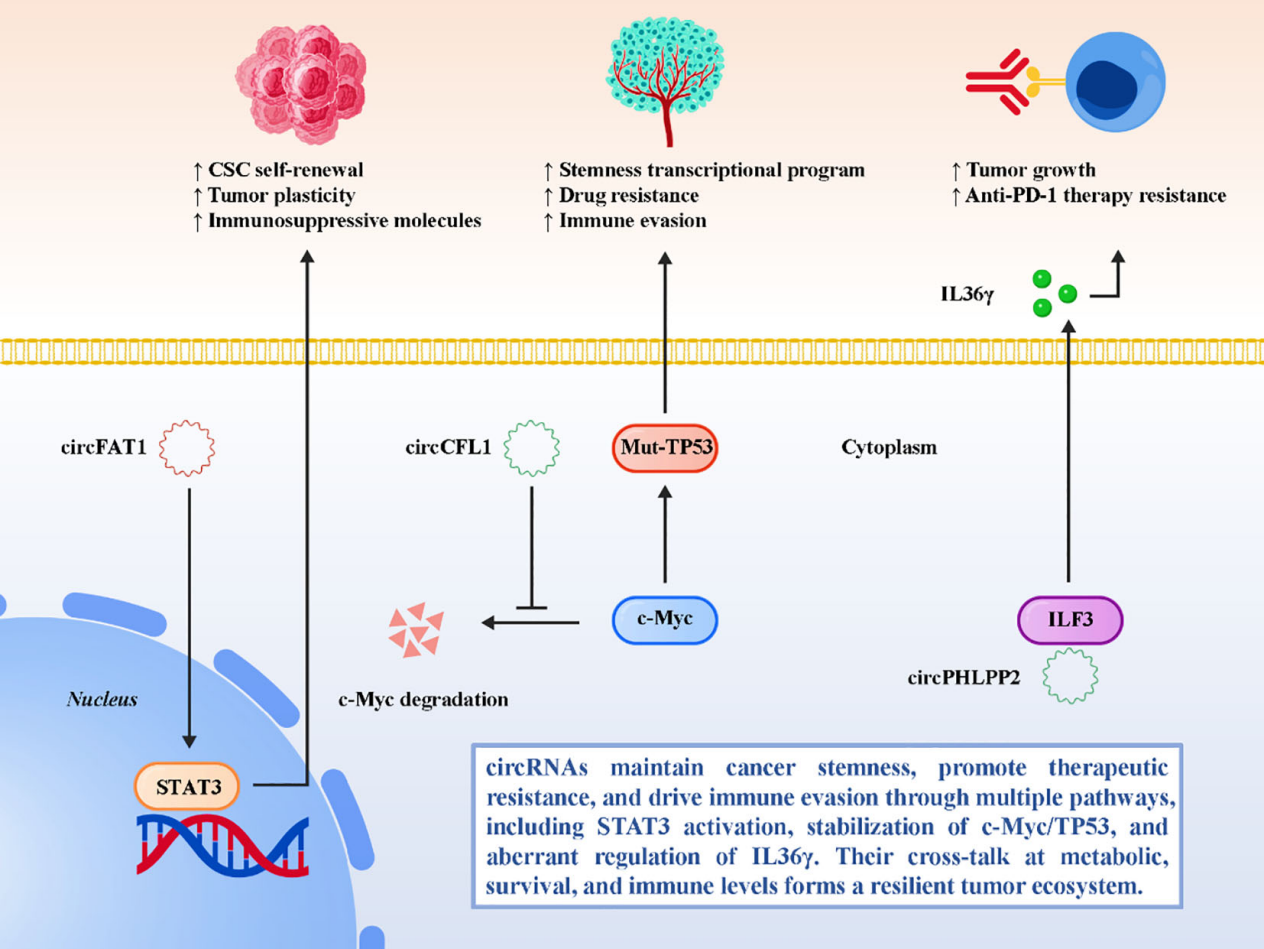
circRNAs link cancer stemness, therapeutic resistance, and immune escape to maintain a malignant ecosystem.

CircRNAs are also intimately linked to the acquisition of cancer stem cell (CSC)–like traits and the development of therapeutic resistance, processes that frequently overlap with immune evasion (63–66). Cancer stemness not only drives tumor initiation and metastasis but also confers a survival advantage under therapeutic stress, while resistant phenotypes often exploit immunosuppressive pathways to persist despite treatment (67–69). Increasing evidence suggests that circRNAs serve as molecular hubs that integrate stemness, resistance, and immune regulation. CircFAT1 exemplifies this interplay by activating the STAT3 signaling pathway, a central regulator of stemness and immune tolerance (42). Through sustained STAT3 activation, circFAT1 promotes CSC-like self-renewal, enhances tumor plasticity, and simultaneously upregulates immunosuppressive mediators that impair cytotoxic T cell function. This dual role reinforces the vicious cycle between stemness and immune escape. Similarly, in triple-negative breast cancer (TNBC), circCFL1 facilitates mutant TP53 transcription by preventing c-Myc degradation through deubiquitylation (43). This mechanism not only stabilizes oncogenic transcriptional programs that underpin stemness and drug resistance but also enhances immune evasion, highlighting the convergent roles of circRNAs in tumor aggressiveness. Another compelling example is circPHLPP2 in colorectal cancer, which interacts with the RNA-binding protein ILF3 to upregulate IL36γ transcription (44). While IL36γ is traditionally associated with proinflammatory signaling, in this context its dysregulated expression paradoxically supports tumor growth and contributes to resistance against anti-PD-1 therapy. This finding emphasizes the context-dependent functions of circRNAs and illustrates how they can subvert immune pathways to sustain resistant phenotypes. Beyond these examples, circRNAs have been implicated in therapy resistance across a wide spectrum of treatments. Certain circRNAs modulate epithelial–mesenchymal transition (EMT), which not only drives metastasis and chemoresistance but also facilitates immune escape by reducing tumor immunogenicity (48, 70–75). Others reprogram autophagy and apoptosis pathways, buffering cancer cells against cytotoxic stress while suppressing immune-mediated cell death. These overlapping layers of regulation create resilient tumor ecosystems that are difficult to eradicate with monotherapies. In summary, circRNAs contribute to cancer stemness and therapeutic resistance by converging on oncogenic transcriptional regulators, metabolic and survival pathways, and immune evasion mechanisms. By integrating these features, circRNAs act as key nodes that maintain tumor heterogeneity and plasticity while sustaining immune suppression. This complexity underscores the therapeutic challenge but also identifies circRNAs as attractive targets for combination strategies aimed at simultaneously disrupting stemness, overcoming resistance, and restoring effective antitumor immunity. **Figure 4** depicts the integrative role of circRNAs in coupling cancer stemness with therapeutic resistance and immune escape.

### 2.5 circRNA regulation of CAR-T cell function, persistence, and exhaustion

Adoptive cell therapies such as chimeric antigen receptor T-cell (CAR-T) therapy have markedly improved outcomes in hematologic malignancies; however, their efficacy in solid tumors remains limited due to T-cell exhaustion, poor persistence, and the highly immunosuppressive tumor microenvironment (76, 77). Increasing evidence indicates that circRNAs are important regulators of T-cell biology and may influence multiple aspects of CAR-T cell function, including activation, metabolic adaptation, cytokine production, and exhaustion programs. Although this field is still in an early developmental stage, current studies suggest that circRNAs exert critical effects on CAR-T therapeutic performance and offer new avenues for engineering next-generation cellular therapies (78, 79).

One of the most promising directions is the integration of circRNA technologies into CAR-T cell engineering. CircRNAs possess unique biochemical advantages—including exceptional stability, resistance to exonuclease degradation, and more sustained protein expression compared with traditional mRNA constructs. These properties make circRNAs attractive tools for improving CAR-T durability and functional persistence. For example, a recent study on DLL3-targeted CAR-T cells for small-cell lung cancer (SCLC) demonstrated that circRNA-based CAR constructs enhance CAR expression stability, alleviate early T-cell exhaustion, and increase tumor-killing capacity, suggesting that circRNA-CAR designs may outperform conventional mRNA-CAR approaches (80). Another study further reported that circRNA-mediated CAR expression can improve antigen specificity and reduce off-target cytotoxicity through more precise transcriptional control, thereby enhancing therapeutic durability and safety (79). In addition, the comprehensive review by Huang et al. summarized the roles of circRNAs in CAR-T cell exhaustion, memory formation, and antigen sensitivity. Their review highlighted that inhibiting exhaustion-associated circRNAs or engineering synthetic circRNAs to boost metabolic fitness could improve CAR-T persistence and long-term activity (77). Notably, synthetic circRNAs with immunostimulatory structures—such as RIG-I-activating viral-like motifs—or those encoding immunomodulatory peptides could also be incorporated into CAR-T manufacturing workflows to further enhance antitumor efficacy. Beyond tuning CAR expression itself, circRNAs may also play broader roles in optimizing CAR-T cell functional performance. By modulating immune signaling pathways—such as IFN-γ production, metabolic adaptation, and activation thresholds—engineered circRNAs have the potential to strengthen CAR-T proliferation, survival, and resilience within the suppressive tumor microenvironment. Moreover, synthetic immunostimulatory circRNAs have been proposed as tools to reshape CAR-T cells toward memory-like phenotypes with reduced exhaustion, a strategy that may help overcome the longstanding barriers to CAR-T efficacy in solid tumors.

Taken together, circRNAs are increasingly recognized as key regulators of CAR-T cell persistence, exhaustion, and tumor specificity. Integrating circRNA biology into CAR-T engineering—such as suppressing exhaustion-promoting circRNAs or delivering immunostimulatory therapeutic circRNAs—may provide powerful strategies for developing more potent, durable, and solid-tumor-compatible next-generation CAR-T cell therapies.

## 3 circRNAs as sensitizers of immunotherapy

While numerous circRNAs contribute to immune suppression, an equally intriguing aspect of circRNA biology is their capacity to enhance antitumor immunity and sensitize tumors to immunotherapy. These “pro-immunogenic” circRNAs act by activating innate immune signaling pathways, inducing immunogenic cell death, and reversing therapeutic resistance, thereby providing novel opportunities to improve the efficacy of ICIs and other immunotherapies (81–83).

### 3.1 Pro-immunogenic circRNAs and innate immune activation

**Figure 5:**
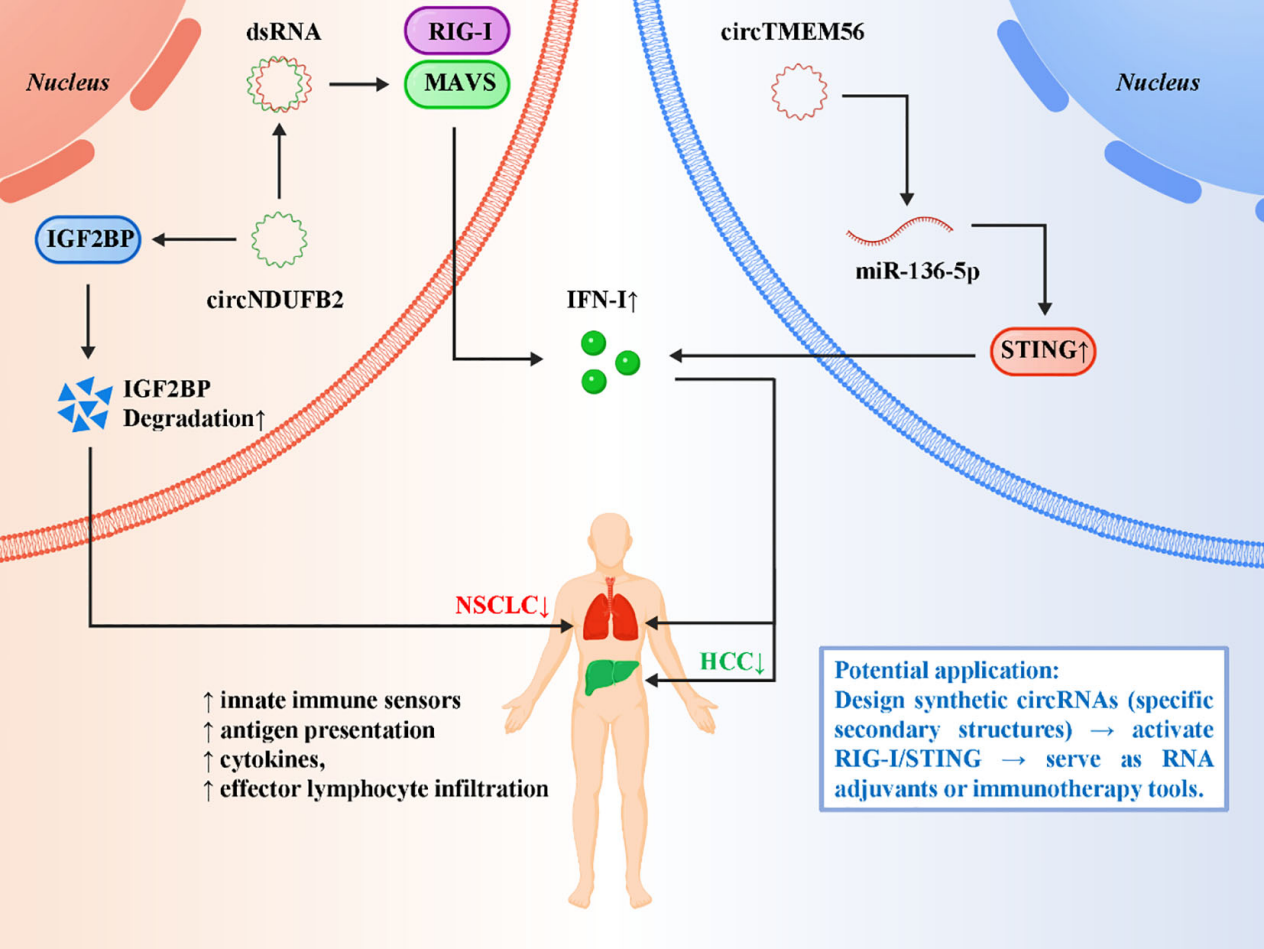
Pro-immunogenic circRNAs activate innate immune pathways to enhance antitumor immunity.

Although many circRNAs function as oncogenic drivers, an emerging subset has been shown to exert tumor-suppressive effects by activating innate immune pathways, thereby sensitizing tumors to immunotherapy. Unlike circRNAs that stabilize PD-L1 or foster immunosuppressive microenvironments, these pro-immunogenic circRNAs act as intrinsic immune activators, often by engaging cytosolic nucleic acid sensors or modulating RNA-binding proteins. A representative example is circNDUFB2 in NSCLC (84). CircNDUFB2 destabilizes oncogenic IGF2BP family proteins, which normally protect tumor-promoting transcripts, thereby reducing oncogenic signaling (84). More importantly, circNDUFB2 forms double-stranded RNA structures that can be recognized by RIG-I, activating the RIG-I–MAVS pathway. This triggers robust type I interferon responses, enhances antigen presentation, and facilitates the recruitment of effector T cells and NK cells into the tumor microenvironment. This dual action not only suppresses tumor growth directly but also reshapes the immune contexture to favor antitumor immunity. Another compelling example comes from hepatocellular carcinoma (HCC), where exosomal circTMEM56 regulates the miR-136-5p/STING axis (28). By relieving repression of STING, circTMEM56 enhances cytosolic DNA sensing and downstream production of interferons and proinflammatory cytokines (28). This pathway amplifies the immunogenic effects of radiotherapy, which generates cytoplasmic DNA fragments, thereby synergizing with existing treatments and augmenting antitumor immunity. Importantly, this example demonstrates how circRNAs can integrate with therapeutic interventions to heighten immune activation. These findings broaden the conceptual framework of circRNA biology by positioning circRNAs not only as passive modulators but also as active initiators of innate immune signaling. Their ability to activate RIG-I or STING pathways mirrors the immunostimulatory properties of viral RNAs, suggesting that endogenous circRNAs may act as “danger signals” when appropriately structured or expressed (85–87). From a translational perspective, this raises the exciting possibility of designing synthetic circRNAs with defined secondary structures to deliberately activate innate immune receptors and function as RNA-based adjuvants for cancer immunotherapy or vaccines. Collectively, these insights reveal that pro-immunogenic circRNAs serve as endogenous immune activators that can potentiate innate immunity and synergize with immune checkpoint blockade. Their unique biology not only expands our understanding of RNA–immune crosstalk but also highlights their translational promise as both biomarkers of immunotherapy responsiveness and templates for engineered RNA therapeutics. **Figure 5** illustrates the emerging role of pro-immunogenic circRNAs in activating innate immune signaling and reshaping the tumor microenvironment.

### 3.2 Induction of immunogenic cell death

**Figure 6:**
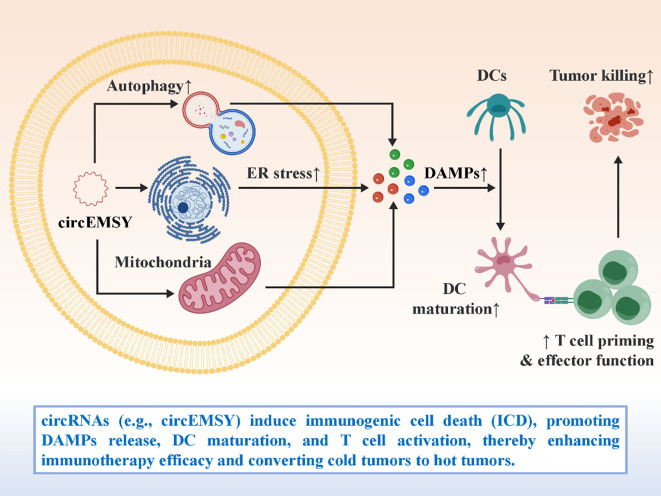
circRNAs induce immunogenic cell death (ICD) to enhance antitumor immunity.

Another emerging function of circRNAs in cancer immunity is their ability to promote ICD, a form of cell death that activates rather than suppresses immune responses. Unlike apoptotic or necrotic death, ICD is characterized by the release and exposure of damage-associated molecular patterns (DAMPs), such as calreticulin (CRT) exposure on the cell surface, extracellular release of ATP, and secretion of high-mobility group box 1 (HMGB1) (88–90). These signals act as potent immunological cues that facilitate dendritic cell (DC) maturation, enhance antigen cross-presentation, and prime cytotoxic CD8^+^ T cell responses against tumor-associated antigens. In lung adenocarcinoma, circEMSY has been identified as a critical regulator of ICD (91). By promoting cellular stress responses and enhancing the immunogenicity of tumor cells, circEMSY facilitates the release of DAMPs that recruit and activate antigen-presenting cells. The resulting amplification of T cell priming significantly boosts the efficacy of PD-1 blockade therapy in preclinical models. This finding provides compelling evidence that circRNAs can convert immunologically “cold” tumors—those with sparse immune infiltration and low antigenicity—into “hot” tumors that are more responsive to immune checkpoint blockade. The ability of circRNAs to modulate ICD has profound therapeutic implications. On one hand, it suggests that endogenous circRNAs may function as natural immunoadjuvants within the tumor microenvironment. On the other, it raises the possibility of therapeutically engineering circRNAs to deliberately induce ICD, thereby enhancing tumor immunogenicity. For example, synthetic circRNAs could be designed to activate stress pathways, increase endoplasmic reticulum stress, or modulate autophagy-related signaling, all of which are closely linked to ICD induction. Such strategies could be combined with checkpoint inhibitors, radiotherapy, or chemotherapy—treatments known to trigger ICD—to synergistically amplify antitumor immunity. Moreover, circRNA-mediated ICD could help overcome one of the major limitations of immunotherapy: the lack of response in tumors with low baseline immune infiltration. By boosting antigen release and dendritic cell activation, circRNA-induced ICD has the potential to expand the pool of patients who benefit from ICIs. In this sense, circRNAs not only serve as biomarkers of immunogenicity but also represent candidate therapeutic tools to reshape the immunological landscape of tumors (92–96). **Figure 6** illustrates the immunostimulatory function of circRNAs in driving ICD. In summary, circRNAs capable of inducing ICD exemplify a new layer of RNA-based immunoregulation. By bridging tumor cell death with immune activation, they provide novel opportunities to enhance the efficacy of existing immunotherapies and to design next-generation RNA therapeutics with adjuvant properties.

### 3.3 CircRNAs counteracting therapy resistance and enhancing sensitivity

**Figure 7:**
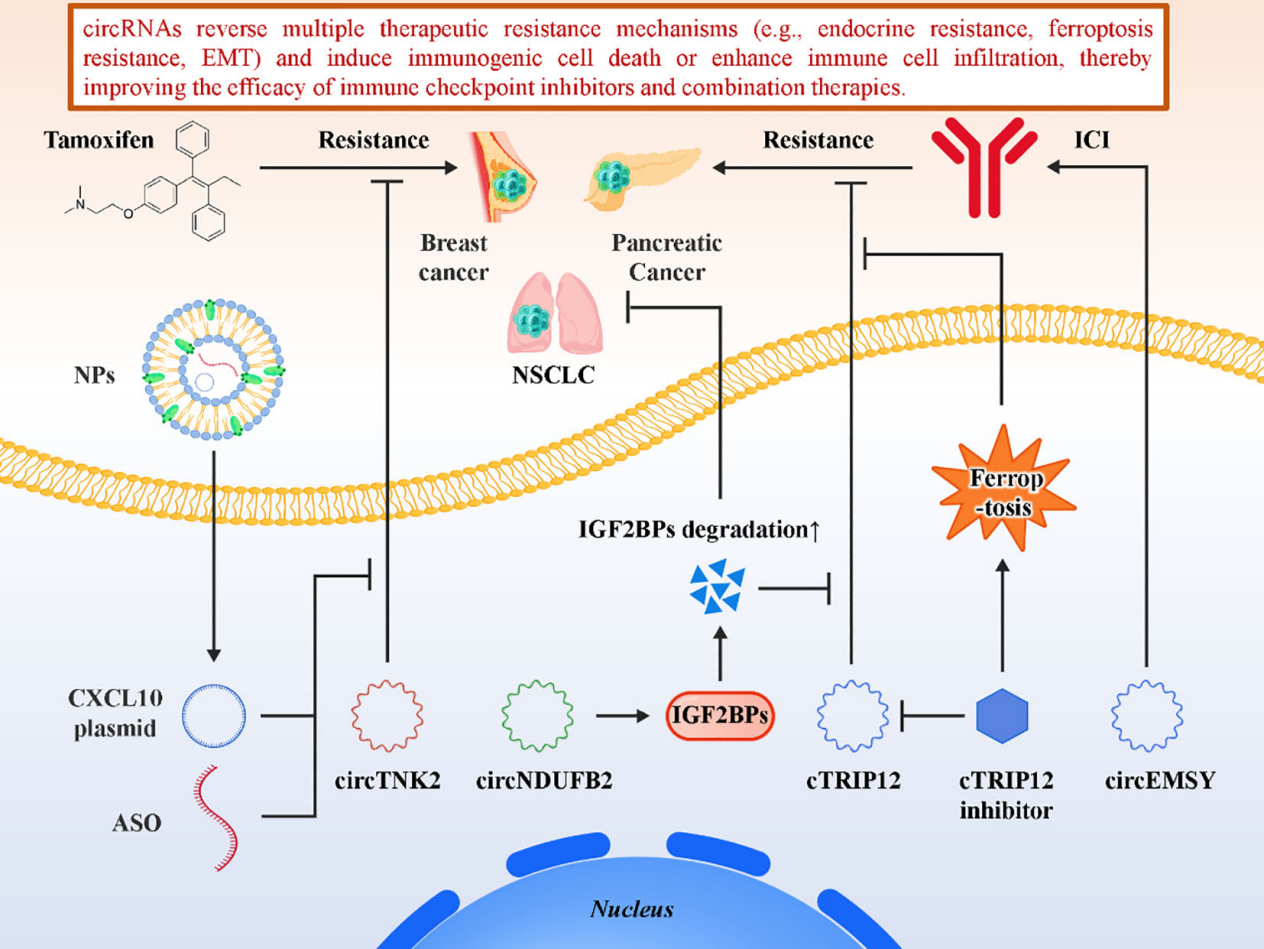
circRNAs reverse therapeutic resistance and enhance immune sensitivity.

Beyond direct activation of immune responses, circRNAs also play a pivotal role in modulating therapeutic resistance, thereby indirectly sensitizing tumors to immunotherapy (75, 97–100). Resistance to conventional therapies—including endocrine therapy, chemotherapy, targeted therapy, and ferroptosis inducers—often overlaps with mechanisms of immune escape, such as altered signaling pathways, metabolic rewiring, or impaired antigen presentation. CircRNAs, through their ability to fine-tune gene expression networks, can either reinforce or dismantle these resistance programs, ultimately shaping tumor responsiveness to immune checkpoint blockade. In estrogen receptor–positive (ER^+^) breast cancer, a notable example is circTNK2, which has been therapeutically targeted using nanoparticles co-loaded with antisense oligonucleotides (ASOs) and immunomodulatory CXCL10 plasmids (101). This combinatorial approach not only restored tamoxifen sensitivity by silencing oncogenic circTNK2 but also enhanced natural killer (NK) cell infiltration and cytotoxicity within the tumor microenvironment. These findings provide compelling proof-of-concept that circRNA modulation can be integrated with standard endocrine therapy to reverse resistance and simultaneously boost antitumor immunity. In pancreatic cancer, targeting cTRIP12 was reported to overcome ferroptosis resistance, a newly recognized cell death resistance pathway associated with poor immunotherapy response (102). By restoring ferroptotic sensitivity, cTRIP12 targeting increased tumor vulnerability to immune checkpoint therapy, highlighting the potential of circRNAs to act at the intersection of metabolic regulation, cell death modalities, and immune reactivation. Additional studies further support the role of circRNAs in therapy sensitization. For example, in non–small cell lung cancer, circNDUFB2 not only destabilizes oncogenic IGF2BP proteins but also enhances immune signaling, thereby synergizing with checkpoint blockade (84). Similarly, engineered delivery of circRNAs capable of inducing immunogenic cell death (e.g., circEMSY) has been shown to sensitize tumors to PD-1/PD-L1 inhibitors by transforming immune-cold tumors into immune-hot ones (91). Collectively, these findings highlight the versatility of circRNAs as mediators of cross-talk between therapeutic resistance and immune responsiveness. From a translational standpoint, circRNAs represent a unique class of immunotherapy sensitizers. By reversing therapy-induced immune resistance—whether through endocrine modulation, ferroptosis reactivation, or restoration of immunogenicity—circRNAs expand the therapeutic repertoire available for resistant tumors. Their ability to function as both therapeutic targets (e.g., oncogenic circRNAs silenced by ASOs) and therapeutic agents (e.g., synthetic circRNAs delivered to promote immune activation) underscores their dual potential. The incorporation of circRNA modulation into rational combination strategies with checkpoint inhibitors, radiotherapy, or metabolic drugs could therefore overcome resistance barriers and improve patient outcomes. In summary, pro-immunogenic circRNAs provide promising entry points for next-generation combination therapies. By activating innate immunity, inducing immunogenic cell death, and reversing therapy resistance, they represent both biomarkers and functional targets that can be harnessed to broaden the scope and efficacy of cancer immunotherapy. **Figure 7** illustrates the integrative role of circRNAs in reversing therapeutic resistance and enhancing immune sensitivity. **Table 2** summarizes the emerging class of circRNAs that function as positive regulators of antitumor immunity by activating innate immune pathways, inducing immunogenic cell death, or reversing resistance to systemic therapies.

**Table 2:** Mechanisms and representative molecules of circRNAs in immunotherapy sensitization.

Mechanism	circRNA	Specific mechanism	Cancer types
Activate the innate immune pathway	circNDUFB2	Activate the RIG-I – MAVS pathway by forming dsRNA structures, thereby inducing type I interferon response and recruiting effector cells	Non-small cell lung cancer (84)
	circTMEM56	Enhancing DNA perception and interferon production through miR-136-5p/STING axis, this mechanism can synergize with radiotherapy	Hepatocellular carcinoma (28)
Inducing immunogenic cell death	circEMSY	Promote the release of DAMP (such as CRT, ATP, HMGB1), enhance dendritic cell activation and T cell initiation	Lung adenocarcinoma (91)
Reverse treatment resistance	circTNK2	Targeted inhibition can restore tamoxifen sensitivity and enhance NK cell infiltration and cytotoxicity	ER+breast cancer (101)
	cTRIP12	Targeted inhibition can restore iron death sensitivity, thereby enhancing the efficacy of immune checkpoint therapy	Pancreatic cancer (102)

## 4 Clinical applications of circRNAs in immuno-oncology

### 4.1 Prognostic and diagnostic potential

Several circRNAs have been linked to patient outcomes and immune microenvironment characteristics, underscoring their value as prognostic and diagnostic indicators in oncology. Owing to their covalently closed structure and resistance to exonuclease degradation, circRNAs are highly stable in both tissue and circulation, making them especially suitable for clinical biomarker development (51, 103–106). Unlike many protein-based markers, circRNAs can reflect both tumor-intrinsic pathways and the status of the TIME, thereby offering a dual layer of biological insight. In gastric cancer, hsa_circ_0072309 has been identified as a prognostic biomarker, with high expression levels correlating with immune cell infiltration and poorer overall survival (107). This suggests its potential utility in predicting disease progression and stratifying patients for more aggressive therapeutic interventions. Likewise, hsa_circ_0001479 and circ_0008287 have been linked to enhanced immune escape, stemness features, and tumor aggressiveness, further highlighting their role as both diagnostic and prognostic markers (41, 108). Beyond gastric cancer, other tumor types also exhibit circRNAs with prognostic significance. In colorectal cancer, circ_0089761 promotes metastasis and immune escape through the miR-27b-3p/PD-L1 axis, and its elevated expression is associated with advanced disease stage and reduced survival, suggesting its potential as a marker of poor prognosis (10). In breast cancer, circ_0067842 facilitates metastasis and immune evasion via the HuR/CMTM6/PD-L1 axis, with its expression levels correlating with aggressive phenotypes and worse clinical outcomes (45). Similarly, in lung cancer, several circRNAs including circIGF2BP3 and circNDUFB2 have been linked to differential immune responses, with the former promoting resistance to immune checkpoint inhibitors and the latter associated with enhanced antitumor immunity, making them potential predictive biomarkers for immunotherapy responsiveness (34, 46, 109). Moreover, circRNAs detected in serum and exosomes offer minimally invasive tools for early detection and disease monitoring. For example, tumor-derived exosomal circRNAs such as circUSP7 and circ_0001947 not only reflect the immunological state of the tumor but also correlate with clinical resistance to PD-1 blockade, suggesting their potential as circulating predictive markers (27, 37, 39). Such liquid biopsy approaches could provide real-time assessment of tumor progression, immune evasion, and therapeutic response, complementing traditional histopathology. Taken together, these studies demonstrate that circRNAs hold significant promise as diagnostic and prognostic biomarkers. Their correlation with immune infiltration, disease aggressiveness, and therapy resistance highlights their potential utility in guiding patient management, from risk stratification to treatment selection. As clinical validation expands, circRNAs may complement or even surpass existing biomarkers in precision oncology, particularly in the era of immunotherapy.

### 4.2 Predictive biomarkers for immunotherapy response

An emerging and clinically relevant area of circRNA research is their potential use as predictive biomarkers of immunotherapy responsiveness. Unlike prognostic biomarkers that reflect the natural course of disease, predictive biomarkers can guide therapeutic decision-making by identifying patients most likely to benefit from specific treatments such as ICIs (110–115). CircRNAs are particularly attractive in this context because they capture tumor-intrinsic signaling as well as cross-talk with the immune microenvironment, and exosomal circRNAs offer a window into these processes through liquid biopsy. Exosomal circRNAs have been especially informative because they reflect real-time communication between tumor and immune cells (51, 52, 116, 117). In NSCLC, exosomal circUSP7 was found to induce CD8^+^ T cell dysfunction through the miR-934/SHP2 axis, thereby driving resistance to PD-1 blockade (37). Its detection in circulating vesicles suggests that patients with elevated exosomal circUSP7 may exhibit poor responses to ICI therapy, making it a promising predictive marker. Similarly, in gastric cancer, exosomal circ_0001947 was reported to promote CD8^+^ T cell exhaustion and confer resistance to PD-1 inhibitors, highlighting the role of circRNAs in shaping acquired resistance to immunotherapy (27). In bladder cancer, circZNF609 enhances fatty acid uptake via the IGF2BP2/CD36 pathway, leading to metabolic reprogramming that reduces tumor sensitivity to immunotherapy (40). This study demonstrates how circRNAs can predict immune checkpoint efficacy not only through immune modulation but also by controlling metabolic pathways that indirectly impair immune surveillance. Other examples include circ_0010235 and circ_0089761, which regulate PD-L1 expression in lung and colorectal cancers, respectively, and have been associated with poor response to checkpoint inhibition, suggesting a more general role for circRNAs in modulating therapeutic outcomes (10, 118). Beyond resistance, circRNAs may also help identify patients more likely to respond to ICIs. For instance, the tumor-suppressive circRNA circNDUFB2, which activates the RIG-I–MAVS innate immune pathway, has been correlated with enhanced interferon signaling and immune cell infiltration in NSCLC, implying that its expression could predict favorable responses to immunotherapy (119). Similarly, circRNAs that promote immunogenic cell death (e.g., circEMSY) or stimulate the STING pathway (e.g., circTMEM56) may serve as biomarkers of “immune-hot” tumors more amenable to checkpoint blockade (28, 91). Collectively, these studies illustrate that circRNAs have dual potential as negative and positive predictive biomarkers: some indicate resistance by stabilizing PD-L1 or fostering immunosuppressive microenvironments, while others point to enhanced sensitivity through activation of innate immune pathways. Their stability in body fluids and availability via noninvasive sampling make them especially suitable for liquid biopsy–based companion diagnostics, enabling dynamic monitoring of treatment responses. As immunotherapy moves toward personalized medicine, integrating circRNA signatures with existing biomarkers such as PD-L1 expression, tumor mutational burden, and T cell receptor repertoire may improve patient stratification and optimize therapeutic outcomes.

### 4.3 Liquid biopsy and clinical applications

The enrichment of circRNAs in circulating exosomes and other extracellular vesicles provides a strong foundation for their development as liquid biopsy biomarkers. Unlike tissue biopsies, which are invasive and may not fully capture tumor heterogeneity, liquid biopsies offer a minimally invasive means of repeatedly sampling the systemic circulation (120–126). CircRNAs are particularly suited to this role due to their covalently closed loop structure, which renders them resistant to exonuclease-mediated degradation and ensures remarkable stability in serum, plasma, saliva, and even urine. These properties allow circRNAs to remain detectable at high fidelity, making them reliable candidates for routine clinical monitoring. Functionally, circulating circRNAs can be exploited to monitor tumor immune status, track treatment response, and detect early signs of therapeutic resistance. For example, exosomal circUSP7 and circ_0001947 have been associated with resistance to PD-1 blockade in NSCLC and gastric cancer, respectively, suggesting their use as early warning markers of immunotherapy failure (27, 37). Similarly, circZNF609 in bladder cancer has been linked to reduced immunotherapy sensitivity through lipid metabolic reprogramming, underscoring how circulating circRNAs can mirror tumor–immune dynamics in real time (40). Conversely, the presence of immunostimulatory circRNAs such as circNDUFB2 or circTMEM56 might indicate a more favorable immune landscape and predict responsiveness to checkpoint inhibitors (28, 84). Beyond response prediction, circRNAs offer opportunities for patient stratification. Their distinct expression profiles across cancer types and immune subgroups could help identify patients most likely to benefit from specific therapies, enabling precision immunotherapy strategies. For instance, patients with high exosomal circUSP7 might be considered for alternative strategies such as NK cell therapy or bispecific antibodies, while those with high pro-immunogenic circRNAs could be prioritized for checkpoint blockade. From a translational perspective, circRNAs also hold promise as companion diagnostics (127–129). Integrating circRNA expression with established biomarkers such as PD-L1 immunohistochemistry, tumor mutational burden, and microsatellite instability may improve predictive accuracy and overcome limitations of single-marker approaches. Moreover, multiplex circRNA profiling through next-generation sequencing or digital droplet PCR could allow comprehensive immune monitoring with relatively low sample requirements (130–134). Despite these advantages, challenges remain. Standardization of detection methods, including protocols for exosome isolation, circRNA quantification, and normalization, will be critical to ensure reproducibility across laboratories. Additionally, the clinical significance of specific circRNAs must be validated in large, prospective, and well-annotated patient cohorts, ideally embedded in immunotherapy clinical trials. Addressing these issues will be key to moving circRNA-based liquid biopsy tests from bench to bedside. In summary, circRNAs are emerging as robust, stable, and clinically relevant biomarkers in cancer immunology. Their prognostic, diagnostic, and predictive value—particularly in the context of immunotherapy response—supports their development as liquid biopsy tools and companion diagnostics. With continued technological improvements and clinical validation, circRNA-based assays may soon complement or even surpass current biomarker strategies, enabling dynamic and personalized monitoring of immunotherapy in clinical practice.

### 4.4 Therapeutic targeting of oncogenic circRNAs

Oncogenic circRNAs that promote immune suppression represent a new class of therapeutic vulnerabilities in cancer. CircRNAs such as circUSP7, circRHBDD1, and circ_0001947 have been shown to drive CD8^+^ T cell dysfunction, stabilize PD-L1, or induce T cell exhaustion, making them compelling targets for interventions aimed at restoring effective antitumor immunity (27, 35, 37). Unlike protein-coding oncogenes, circRNAs are defined by their unique back-splice junctions, which enable the design of highly specific silencing strategies with minimal off-target effects on linear transcripts. Current approaches for circRNA targeting primarily include antisense oligonucleotides (ASOs) and small interfering RNAs (siRNAs) (135–138). These molecules are engineered to hybridize with the back-splice junction sequences of circRNAs, leading to their selective degradation. Preclinical studies have provided proof-of-concept for this strategy. For example, nanoparticle-delivered ASOs targeting circRHBDD1 in gastric cancer reduced PD-L1 expression and enhanced CD8^+^ T cell cytotoxicity, thereby sensitizing tumors to immune checkpoint blockade (35). Similarly, silencing circUSP7 in NSCLC disrupted exosomal signaling that drives T cell exhaustion and restored responsiveness to anti-PD-1 therapy. These findings suggest that circRNA silencing can not only suppress tumor-intrinsic pathways but also reprogram the immune microenvironment. Therapeutic targeting of exosomal circRNAs also presents unique opportunities. Since exosomal circRNAs such as circ_0001947 and circRNA_0013936 mediate immune suppression by modulating T cell and MDSC activity, their inhibition may dismantle immunosuppressive networks across local and systemic compartments (27, 38). Importantly, targeting exosomal circRNAs could complement strategies aimed at tumor-intrinsic circRNAs, offering a more comprehensive reactivation of immune surveillance (110, 111, 139–141). Nanotechnology further enhances the feasibility of circRNA-based therapeutics. Nanoparticles, lipid carriers, and polymeric systems have been employed to deliver ASOs or siRNAs with improved tumor targeting, stability, and reduced systemic toxicity (142). For instance, PLGA-PEG nanoparticles and lipid nanoparticles (LNPs)—already validated in the delivery of mRNA vaccines—are being adapted for circRNA-targeting strategies, underscoring their clinical translational potential (143). Despite these advances, challenges remain. CircRNAs often function within complex regulatory networks, and targeting a single circRNA may lead to compensatory mechanisms. Furthermore, ensuring the specificity of junction-targeted ASOs is critical to avoid unintended silencing of linear transcripts. Off-target immune stimulation and delivery efficiency must also be carefully evaluated in preclinical studies. In summary, oncogenic circRNAs that sustain immune suppression represent promising therapeutic targets. Advances in ASO and siRNA design, coupled with nanoparticle-based delivery systems, have provided strong preclinical proof-of-concept for circRNA-targeted therapy. Moving forward, the integration of circRNA silencing strategies with immune checkpoint inhibitors, metabolic reprogramming agents, or radiotherapy may offer a synergistic approach to recondition the TIME and enhance clinical outcomes.

### 4.5 Engineering pro-immunogenic circRNAs

In addition to silencing oncogenic circRNAs, an equally promising therapeutic strategy is to introduce or enhance circRNAs with pro-immunogenic functions. This approach leverages the unique structural and functional properties of circRNAs to reprogram the tumor microenvironment and augment immune responses (144). Compared with linear RNAs, synthetic circRNAs display enhanced stability, prolonged half-life, and reduced innate degradation, making them particularly attractive as therapeutic agents. Engineered circRNAs can be designed to activate innate immune pathways or to act as stable mimics of tumor-suppressive circRNAs. For example, delivery of circNDUFB2 not only destabilizes oncogenic IGF2BPs but also directly activates the RIG-I–MAVS signaling pathway, leading to robust type I interferon production and recruitment of immune effector cells (84). This dual action has the potential to both weaken tumor cell fitness and strengthen immune surveillance. Similarly, exogenous expression of circEMSY has been shown to trigger ICD in lung adenocarcinoma, resulting in increased antigen exposure, dendritic cell activation, and improved efficacy of PD-1 blockade (91). These findings provide proof-of-principle that therapeutic circRNAs can convert immunologically “cold” tumors into “hot” tumors, thereby expanding the population of patients responsive to checkpoint inhibitors. Advances in RNA engineering technologies further enhance the therapeutic potential of circRNAs. Synthetic circRNAs can be optimized with specific secondary structures that mimic viral RNA, thereby potentiating recognition by innate sensors such as RIG-I or MDA5 (84–86, 145–149). Alternatively, circRNAs can be engineered to harbor functional RNA elements or small open reading frames (sORFs), allowing them to regulate protein translation or encode immunomodulatory peptides (150–152). These design strategies extend the scope of circRNAs from passive regulators to active immunotherapeutic molecules. From a translational perspective, engineered circRNAs could be delivered systemically or locally using lipid nanoparticles (LNPs), polymeric carriers, or viral vectors. LNP platforms, which have already demonstrated clinical success in mRNA vaccines, can be adapted to encapsulate and deliver circRNAs efficiently to tumor tissues. Moreover, combining engineered circRNAs with existing therapies—such as checkpoint inhibitors, radiotherapy, or chemotherapy—could create synergistic effects by simultaneously enhancing immune activation and disrupting tumor survival pathways. In summary, engineering pro-immunogenic circRNAs represents a novel paradigm in RNA-based therapy. By mimicking or amplifying natural tumor-suppressive circRNAs, synthetic constructs can activate innate immunity, induce immunogenic cell death, and sensitize tumors to immunotherapy. This strategy expands the functional repertoire of RNA therapeutics, offering a versatile and durable platform for next-generation cancer immunotherapy.

### 4.6 Nanomedicine and drug delivery systems

Efficient and tumor-specific delivery remains one of the most critical challenges for circRNA-based therapies. Naked RNA molecules are inherently unstable in the circulation and susceptible to rapid degradation by nucleases, while systemic administration risks off-target effects and poor accumulation within tumors (33, 153–156). Nanomedicine platforms have emerged as powerful solutions to overcome these limitations by providing stability, targeted delivery, and the possibility of co-delivering therapeutic agents to modulate both tumor-intrinsic and immune pathways. Several classes of nanocarriers have been evaluated in preclinical models. PLGA-PEG nanoparticles have been used to encapsulate circRNA inhibitors, protecting them from enzymatic degradation and improving tumor accumulation in gastric cancer models, where they successfully reduced PD-L1 expression and enhanced T cell–mediated killing. ZIF-8 nanoparticles, a subclass of metal–organic frameworks (MOFs), have shown high loading capacity and pH-responsive release properties. In estrogen receptor–positive breast cancer, ZIF-8 nanoparticles were employed to co-deliver antisense oligonucleotides (ASOs) targeting circTNK2 and plasmids encoding CXCL10 (101, 157). This combinatorial approach not only restored tamoxifen sensitivity but also recruited natural killer (NK) cells into the tumor microenvironment, demonstrating how nanocarriers can achieve synergistic immunomodulation. In addition, lipid nanoparticles (LNPs)—already clinically validated for the delivery of mRNA vaccines against SARS-CoV-2—are particularly attractive for circRNA therapeutics (158–160). Their biocompatibility, scalable production, and ability to protect RNA cargo while enabling endosomal escape make them a clinically feasible platform for systemic circRNA delivery. LNP formulations can be further modified with tumor-targeting ligands (e.g., antibodies, peptides, aptamers) to enhance tissue specificity and minimize off-target effects. Future nanomedicine approaches could integrate circRNA modulation with other therapeutic modalities. For example, nanoparticles may be engineered for co-delivery of circRNA inhibitors with immune checkpoint inhibitors, enabling simultaneous reactivation of T cell function and disruption of oncogenic signaling. Alternatively, combining circRNA-targeting agents with radiotherapy or metabolic inhibitors may enhance immunogenic cell death and metabolic reprogramming, creating a more favorable TIME (161). Smart nanocarriers with stimuli-responsive release mechanisms (e.g., pH, hypoxia, ROS-sensitive systems) are also under development to ensure precise delivery in the hostile tumor milieu while reducing systemic toxicity. Despite these advances, challenges remain for clinical translation. Nanocarriers must balance delivery efficiency, biocompatibility, and immunogenicity, and long-term safety data are limited. Additionally, the complexity of manufacturing RNA-loaded nanoparticles at scale under good manufacturing practice (GMP) conditions remains a significant hurdle. Nevertheless, rapid progress in nanomedicine, exemplified by the global rollout of RNA vaccines, underscores the feasibility of adapting similar platforms for circRNA-based therapeutics. In summary, nanomedicine offers a transformative solution for circRNA delivery, enabling stable, targeted, and multifunctional therapeutic strategies. By combining circRNA modulation with checkpoint blockade, radiotherapy, or metabolic therapies, nanoparticle platforms have the potential to recondition the tumor microenvironment and expand the clinical utility of RNA-based cancer immunotherapies. **Table 3** summarizes the expanding clinical relevance of circRNAs, highlighting their multifaceted roles as biomarkers, therapeutic predictors, and direct intervention targets in cancer immunotherapy.

**Table 3:** Clinical application prospects of circRNAs.

Application	circRNA	Clinical significance	Cancer types
Prognostic and diagnostic biomarkers	hsa_circ_0072309	Prognostic markers of gastric cancer, high expression associated with immune infiltration and poor overall survival	Gastric cancer (107)
	circ_0089761	Promoting colorectal cancer metastasis and immune escape through miR-27b-3p/PD-L1 axis, indicating poor prognosis	Colorectal cancer (10)
	circ_0067842	Promote breast cancer metastasis and immune escape through HuR/CMTM6/PD-L1 axis	Breast cancer (45)
Immunotherapy predictive biomarkers	exosomal circUSP7	Predicting resistance to PD-1 therapy	Non-small cell lung cancer (37)
	exosomal circ_0001947	Predicting resistance to PD-1 therapy	Gastric cancer (27)
	circZNF609	Predicting decreased sensitivity of immunotherapy through metabolic reprogramming	Bladder cancer (40)
	circNDUFB2	Predicting the response of tumor immunotherapy	Non-small cell lung cancer (84)
therapeutic target	circRHBDD1	ASO targeting circRHBDD1 can reduce PD-L1 and enhance T cell killing	Gastric cancer (35)
	circUSP7	Targeting circUSP7 can increase the number of T cells	Non-small cell lung cancer (37)
Engineering therapy molecules	Engineering circNDUFB2	As a therapeutic agent, it can activate the RIG-I pathway and enhance immune response	(84)
	Engineering circEMSY	As a therapeutic agent, it can induce ICD and enhance the efficacy of PD-1	(91)

## 5 Challenges and future directions

Despite the rapid progress in uncovering circRNA functions in tumor immunity, significant challenges remain in translating these discoveries into reliable clinical applications (162–165). The complexity of circRNA biology, methodological limitations, and gaps in clinical validation highlight the need for cautious interpretation and rigorous future research.

### 5.1 Biological complexity and heterogeneity

CircRNAs are inherently diverse in their biogenesis, sequence composition, cellular localization, and functional roles, and this heterogeneity complicates efforts to generalize their biological impact across different tumor contexts. Unlike linear RNAs, circRNAs are generated through alternative back-splicing events, often producing multiple isoforms from the same parental gene. These isoforms can vary in exon composition, length, and secondary structure, leading to distinct molecular interactions and downstream effects. For instance, circRNAs localized predominantly in the cytoplasm may act as microRNA sponges or protein scaffolds, while nuclear-enriched circRNAs often modulate transcription or splicing, resulting in context-specific outcomes (166–171). Adding to this complexity, the same circRNA can exhibit divergent functions depending on tumor type, immune landscape, or even treatment exposure. For example, while certain circRNAs such as circIGF2BP3 or circRHBDD1 stabilize PD-L1 and promote immune escape in lung and gastric cancers, others like circNDUFB2 activate innate immunity via RIG-I signaling and enhance checkpoint blockade efficacy in NSCLC (34, 35, 46). Similarly, circRNAs that promote glycolysis in one tumor type may have negligible or even opposite effects in another, depending on metabolic dependencies and immune cell composition within the tumor microenvironment. This duality reflects the plasticity of circRNA–protein and circRNA–RNA interactions, which are influenced by cell type–specific expression of RNA-binding proteins, microRNAs, and epigenetic states (172–174). Clinical implications of this heterogeneity are significant (120, 175, 176). A circRNA that serves as a biomarker of poor prognosis in one cancer could, in another context, predict better responsiveness to immunotherapy. This variability underscores the importance of context-dependent analyses and suggests that circRNA-targeted interventions must be carefully stratified by cancer type, molecular subtype, and immune phenotype. It also highlights the need for large-scale, multi-omics studies that integrate transcriptomic, proteomic, metabolic, and immunogenomic data to unravel circRNA functions in specific disease contexts. In summary, the biological complexity and heterogeneity of circRNAs pose both challenges and opportunities. They complicate the development of “one-size-fits-all” therapeutic strategies but also provide a basis for highly personalized circRNA-based diagnostics and treatments. Moving forward, mapping circRNA functions across diverse cancer–immune ecosystems will be critical for identifying those with consistent clinical relevance and for tailoring therapeutic approaches to specific tumor settings.

### 5.2 Technical and methodological limitations

Accurate detection and quantification of circRNAs remain one of the major technical hurdles in the field. Unlike linear RNAs, circRNAs lack free 5′ and 3′ ends, making them refractory to standard RNA-seq pipelines that are optimized for linear transcript annotation (163, 177–179). Conventional RNA-sequencing approaches often misclassify circular transcripts as splicing noise or underrepresent them due to reliance on short-read alignments. The identification of back-splice junctions, the defining feature of circRNAs, requires specialized computational algorithms such as CIRCexplorer, find_circ, CIRI2, and DCC. However, these tools can produce discordant results depending on alignment settings, reference genome versions, and filtering thresholds, leading to inconsistent circRNA catalogs across studies. Experimental validation is equally challenging. PCR-based detection methods must be carefully designed with divergent primers spanning the back-splice junction, yet non-specific amplification or contamination with linear isoforms can generate false positives. RNase R digestion, commonly used to enrich for circular species, is not fully specific and can incompletely degrade linear RNAs, leading to overestimation of circRNA abundance. Moreover, circRNAs with short loop lengths or strong secondary structures may resist RNase R digestion, creating further technical bias. Another limitation is the lack of standardized annotation and nomenclature. The same circRNA may be assigned multiple identifiers across databases such as circBase, circAtlas, and circBank, creating confusion and hindering reproducibility. In addition, circRNA expression can be highly cell type– and condition-specific, and many studies report circRNAs without rigorous cross-validation in independent datasets, raising concerns about biological versus technical variability. Quantification remains problematic as well. CircRNAs are typically expressed at lower levels compared with their linear counterparts, making them difficult to detect reliably, especially in liquid biopsy samples such as serum or plasma. Advances in technologies such as nanopore long-read sequencing, single-cell RNA-seq, and droplet digital PCR (ddPCR) offer promising solutions by improving sensitivity and enabling isoform-level resolution. Yet these methods are still limited by cost, throughput, and the need for standardized pipelines. Overall, the technical and methodological limitations of circRNA detection and quantification remain a bottleneck for clinical translation. Standardized bioinformatics pipelines, curated reference datasets, and consensus nomenclature are urgently required to enable cross-study comparability. Furthermore, the integration of multi-omics approaches—combining circRNA profiling with proteomics, metabolomics, and immunogenomics—may help validate functional significance and reduce reliance on a single detection method. Only through rigorous methodological improvements can circRNA research achieve the reproducibility and robustness needed for clinical biomarker development and therapeutic targeting.

### 5.3 Preclinical validation and reproducibility

Most studies investigating circRNA-mediated immune regulation remain at the preclinical stage, relying predominantly on *in vitro* assays and murine xenograft models. While these approaches provide mechanistic insights, they often fail to capture the complexity of the human TIME. Murine models, in particular, may not fully recapitulate human immune cell diversity, cytokine networks, or the heterogeneity of circRNA expression across different cancer subtypes, thereby limiting translational relevance (103, 180–184). A major concern is the small sample sizes commonly used in circRNA studies, which reduce statistical power and increase the risk of type I errors. Variable experimental designs, differences in circRNA detection platforms, and lack of standardized analytical pipelines contribute further to irreproducibility. For example, circRNAs implicated in PD-L1 regulation have been reported across multiple tumor types, yet some of these findings have later been challenged or even retracted, underscoring the importance of stringent quality control, robust experimental design, and independent replication before clinical claims can be substantiated (26, 48, 185–189). Another challenge is that many studies report correlative associations between circRNA expression and immune phenotypes without establishing causality. Functional validation—such as loss- and gain-of-function experiments, rescue assays, and immune cell co-culture systems—is often limited or inconsistent. Moreover, many current studies use xenograft models in immunodeficient mice, which fail to recapitulate the essential role of adaptive immunity in the tumor microenvironment. In contrast, more physiologically relevant models such as syngeneic or humanized immune system mice, which allow for intact tumor–immune interactions, are still underutilized. This methodological gap hampers our ability to accurately evaluate how circRNAs shape antitumor immunity or predict responses to immunotherapy. Technical variability also poses a challenge. Differences in circRNA detection platforms (e.g., RNA-seq versus qRT-PCR), computational pipelines for back-splice junction identification, and poor annotation consistency across studies have resulted in low reproducibility and conflicting findings in the field. Moreover, limited cross-cohort validation and lack of accessible circRNA databases—especially those linked to clinical immunotherapy outcomes—constrain the translational potential of current findings. To overcome these limitations, large-scale, multi-omics profiling is urgently needed. Integrating circRNA transcriptomics with proteomics, metabolomics, and immunogenomics can provide a more holistic understanding of circRNA function and its downstream effectors. Single-cell RNA sequencing and spatial transcriptomics have emerged as powerful tools to map circRNAs at cellular and spatial resolution, offering unprecedented insight into how circRNAs are expressed by specific immune and stromal subsets within the tumor microenvironment. Applying these spatially resolved tools in longitudinal clinical cohorts treated with immunotherapy will shed light on dynamic changes in circRNA expression during immune activation, suppression, or resistance acquisition. In parallel, emerging technologies such as direct RNA sequencing, CRISPR-based circRNA manipulation tools, and high-throughput screening platforms could facilitate functional annotation of circRNA–immune interactions at scale. Coupling these approaches with organoid-based immune co-culture systems or tumor-on-chip models may enable more physiologically relevant and rapid circRNA functional testing compared to traditional animal models. Ultimately, reproducibility and translational success in circRNA research will depend on standardized experimental pipelines, transparent data reporting, and collaborative validation efforts across multiple laboratories. Establishing community-wide circRNA consortia—similar to initiatives like The Cancer Genome Atlas (TCGA) or the Human Cell Atlas—could accelerate progress by generating uniformly processed reference datasets, curated knowledge resources, and coordinated cross-validation networks. Public repositories linking circRNA expression to clinical outcomes, immune profiles, and validated functional annotations will further support independent benchmarking and biomarker development. Only through such rigorous, collaborative, and multi-layered validation can circRNAs transition from intriguing preclinical observations to reliable, clinically actionable biomarkers and therapeutic targets in immuno-oncology. Their dual ability to regulate both tumor cell-intrinsic pathways and the tumor immune landscape underscores their potential as powerful molecular levers in the era of precision immunotherapy.

### 5.4 Delivery and safety concerns in therapeutic development

Although nanomedicine platforms—including lipid nanoparticles (LNPs), polymeric carriers, and metal–organic frameworks—offer promising solutions for circRNA delivery, the systemic manipulation of circRNAs introduces several safety and translational concerns that must be carefully addressed. One major issue is the risk of off-target effects. Junction-specific antisense oligonucleotides (ASOs) and siRNAs are designed to selectively target circRNAs, but incomplete specificity may inadvertently affect linear isoforms or unrelated transcripts with partial sequence complementarity. Such off-target interactions could disrupt essential cellular pathways, leading to unintended toxicity. Similarly, synthetic circRNAs engineered to mimic viral structures may unintentionally trigger innate immune activation through pattern recognition receptors (PRRs) such as RIG-I, MDA5, or TLR3, potentially leading to systemic inflammation or autoimmunity if not carefully controlled (85, 86, 146, 190–193). Another concern is the potential perturbation of endogenous circRNA networks. Many circRNAs are highly conserved and functionally integrated into normal physiology, including regulation of neuronal plasticity, cardiovascular homeostasis, and innate immunity. Therapeutic silencing or overexpression could therefore disrupt physiological circRNA–miRNA–mRNA axes or interfere with RNA-binding protein availability, raising the possibility of unforeseen side effects beyond the tumor microenvironment (194–199). Pharmacokinetics and biodistribution also remain incompletely understood. While nanoparticles can enhance tumor targeting, they may also accumulate in the liver, spleen, or kidneys, leading to off-tumor exposure. Long-term biodistribution studies are essential to evaluate organ-specific toxicity and to optimize delivery systems for maximal therapeutic index. Moreover, the immune-related adverse events (irAEs) commonly seen with checkpoint inhibitors highlight the need to assess how circRNA therapeutics might synergize—or exacerbate—immune toxicities when used in combination regimens. From a translational perspective, challenges also include scalable manufacturing and regulatory approval. Unlike linear RNA drugs, circRNAs present unique structural complexities that require specialized production methods to ensure stability, purity, and reproducibility. Current GMP-grade platforms for circRNA synthesis and formulation remain underdeveloped compared with those for siRNA or mRNA. Furthermore, regulatory frameworks specific to circRNA-based therapeutics are lacking, which may delay clinical trial initiation and approval. Clear guidelines on quality control, safety testing, and long-term monitoring will be essential for advancing circRNA therapeutics into the clinic. In summary, while circRNAs hold immense therapeutic promise, their safe and effective delivery requires addressing challenges related to off-target activity, immune activation, pharmacokinetics, and manufacturing. Future work should focus on designing tumor-specific delivery systems (e.g., ligand-modified nanoparticles, stimuli-responsive carriers), conducting long-term safety studies, and establishing regulatory pathways that reflect the unique properties of circRNAs. Only by resolving these issues can circRNA-based therapies achieve successful and responsible clinical translation.

### 5.5 Clinical translation challenges and future opportunities

Looking ahead, several avenues offer promising opportunities to advance circRNA research in immuno-oncology. First, integrative multi-omics approaches that combine circRNA profiling with immunogenomics, epigenomics, proteomics, and metabolomics will provide a more holistic view of circRNA–immune interactions. Such integrated analyses could help identify circRNAs that function as nodal regulators of immune escape or therapy response, while also clarifying their relationship with other layers of tumor biology. Single-cell and spatial transcriptomic technologies will further allow circRNA mapping at cellular and tissue resolution, enabling researchers to pinpoint circRNA expression in specific immune subsets, stromal compartments, or tumor niches (200–202). Second, the engineering of synthetic circRNAs with programmable immunomodulatory functions represents an exciting frontier for therapeutic innovation. Synthetic circRNAs could be designed to mimic tumor-suppressive circRNAs, to act as stable “immune adjuvants” activating RIG-I or STING pathways, or even to encode short immunomodulatory peptides through small open reading frames (sORFs). Such designer circRNAs would expand the functional repertoire of RNA therapeutics beyond traditional mRNA and siRNA platforms, paving the way for versatile tools that not only inhibit oncogenic signaling but also recondition the immune microenvironment. Third, exosomal circRNAs represent an attractive avenue for liquid biopsy applications. Their stability in circulation and enrichment in extracellular vesicles make them ideal biomarkers for noninvasive disease monitoring. Circulating circRNA signatures could be used to stratify patients before therapy, track dynamic changes in immune responsiveness, and provide early warning signals of acquired resistance. With further validation, circRNA-based assays may become companion diagnostics for immune checkpoint inhibitors and other immunotherapies, complementing or surpassing existing biomarkers such as PD-L1 expression and tumor mutational burden. Fourth, rational combination strategies hold great potential. CircRNA-targeting antisense oligonucleotides could be paired with checkpoint inhibitors to restore T cell function, or combined with metabolic modulators to alleviate immunosuppressive nutrient competition in the TME. CircRNA engineering could also synergize with radiotherapy or chemotherapy by amplifying immunogenic cell death, thereby converting immunologically “cold” tumors into “hot” ones more responsive to immunotherapy. Such combinatorial approaches may broaden the clinical benefit of immunotherapy and overcome resistance in hard-to-treat cancers. Finally, collaborative clinical trials will be indispensable. Incorporating circRNA endpoints into immuno-oncology studies—through biomarker analysis, companion diagnostic development, or interventional strategies—will be critical to validate their predictive and therapeutic utility. Multi-center trials with large, well-annotated patient cohorts are needed to overcome issues of reproducibility and population heterogeneity. Moreover, regulatory frameworks and industry–academia partnerships must evolve in parallel to facilitate the safe, efficient, and standardized translation of circRNA-based technologies into clinical practice. In summary, the future of circRNA research lies in harnessing their dual roles as regulators of immune suppression and activators of immunotherapy sensitivity. By integrating multi-omics data, engineering synthetic circRNAs, leveraging exosomal biomarkers, and designing rational therapeutic combinations, researchers can unlock new diagnostic and therapeutic opportunities. Collaborative clinical efforts will ultimately determine whether circRNAs can transition from bench to bedside, delivering tangible benefits for cancer patients in the era of precision immuno-oncology.

Although circRNAs display considerable potential as biomarkers and therapeutic targets in cancer immunology, substantial challenges remain before they can be translated into clinical practice. First, delivery strategies remain a major barrier. Most circRNA-targeting approaches rely on antisense oligonucleotides, siRNAs, or nanoparticle-based systems, but issues such as tumor-specific delivery, cellular uptake efficiency, immune activation, and long-term biosafety are still unresolved. The lack of standardized delivery platforms limits the feasibility of circRNA-based therapeutics in clinical settings. Second, clinical trial readiness is insufficient. Only a small number of circRNA-related interventions have entered preclinical validation, and none have reached phase I clinical trials. Critical aspects—including pharmacokinetics, pharmacodynamics, immunogenicity, and optimal dosing—remain largely unexplored. More rigorous *in vivo* validation and regulatory frameworks tailored to RNA therapeutics are urgently needed. Third, biomarker validation is still in its infancy. Although numerous circRNAs correlate with immune phenotypes, therapy response, or clinical outcomes, most findings are based on small cohorts or retrospective analyses. Large-scale, prospective, multi-center cohorts with harmonized detection methods are required to validate circRNA signatures as predictive or prognostic biomarkers. In particular, integrating circRNA profiling with established biomarkers such as PD-L1 expression, tumor mutational burden, or immune cell infiltration may enhance their robustness and clinical utility. Looking forward, future development should focus on constructing standardized pipelines for circRNA quantification, optimizing delivery platforms, conducting systematic toxicity studies, and incorporating circRNA endpoints into immunotherapy trials. These advances will be essential for translating circRNA-based discoveries into clinically meaningful tools that can guide patient stratification, predict therapeutic responses, and develop novel immunomodulatory treatments.

## 6 Conclusion

CircRNAs have emerged as multifaceted regulators of tumor–immune dynamics, exerting both immunosuppressive and immunostimulatory effects within the tumor microenvironment. On the one hand, oncogenic circRNAs reinforce immune evasion by stabilizing PD-L1, driving T cell exhaustion, promoting immunosuppressive cell populations such as MDSCs, Tregs, and M2 macrophages, and rewiring tumor metabolism to deprive effector lymphocytes of nutrients. These functions highlight circRNAs as active contributors to tumor immune escape and therapeutic resistance. On the other hand, tumor-suppressive circRNAs and engineered synthetic circRNAs have been shown to activate innate immune signaling, induce immunogenic cell death, restore ferroptosis sensitivity, and enhance responses to checkpoint blockade. This duality underscores the context-dependent nature of circRNA biology and positions circRNAs as central players in maintaining the balance between tumor progression and immune surveillance. From a translational perspective, circRNAs hold promise as next-generation biomarkers and therapeutic targets. Their exceptional stability and detectability in serum, plasma, and exosomes make them strong candidates for liquid biopsy–based diagnostics, capable of predicting prognosis, stratifying patients for immunotherapy, and monitoring therapeutic responses in real time. Therapeutically, advances in antisense oligonucleotides, RNA interference, CRISPR technologies, and nanomedicine provide novel means to silence oncogenic circRNAs or deliver pro-immunogenic circRNAs with high specificity. The concept of “therapeutic circRNAs” thus expands the armamentarium of RNA-based medicine, offering versatile strategies to recondition the TIME. Nevertheless, substantial challenges remain. Technical barriers in circRNA detection and annotation continue to hinder reproducibility across studies, and preclinical findings often lack validation in large, diverse patient cohorts. Delivery systems must be optimized to ensure tumor-specific targeting, durable expression, and minimal off-target toxicity. Moreover, the complexity and heterogeneity of circRNA biology necessitate context-aware therapeutic approaches rather than one-size-fits-all solutions. Addressing these hurdles will require interdisciplinary collaboration across RNA biology, bioinformatics, immunology, nanotechnology, and clinical oncology, as well as the establishment of standardized pipelines and regulatory frameworks for circRNA-based therapeutics. In conclusion, circRNAs represent both a challenge and an opportunity in cancer immunology. By bridging mechanistic insights with translational applications, circRNA research is poised to reshape the landscape of immuno-oncology. Continued advances in biomarker discovery, therapeutic engineering, and clinical validation may ultimately transform circRNAs into powerful tools for precision medicine, offering innovative biomarkers and RNA-based therapeutics that enhance the efficacy and durability of cancer immunotherapy.

## References

[1] Bray F Ferlay J Soerjomataram I Siegel RL Torre LA Jemal A . Global cancer statistics 2018: GLOBOCAN estimates of incidence and mortality worldwide for 36 cancers in 185 countries. CA: Cancer J Clin. (2018) 68:394–424. doi: 10.3322/caac.21492, PMID: 30207593

[2] Bray F Laversanne M Sung H Ferlay J Siegel RL Soerjomataram I . Global cancer statistics 2022: GLOBOCAN estimates of incidence and mortality worldwide for 36 cancers in 185 countries. CA: Cancer J Clin. (2024) 74:229–63. doi: 10.3322/caac.21834, PMID: 38572751

[3] Cao W Qin K Li F Chen W . Comparative study of cancer profiles between 2020 and 2022 using global cancer statistics (GLOBOCAN). J Natl Cancer Center. (2024) 4:128–34. doi: 10.1016/j.jncc.2024.05.001, PMID: 39282581 PMC11390618

[4] Sung H Ferlay J Siegel RL Laversanne M Soerjomataram I Jemal A . Global cancer statistics 2020: GLOBOCAN estimates of incidence and mortality worldwide for 36 cancers in 185 countries. CA: Cancer J Clin. (2021) 71:209–49. doi: 10.3322/caac.21660, PMID: 33538338

[5] Köksal H Müller E Inderberg EM Bruland Ø Wälchli S . Treating osteosarcoma with CAR T cells. Scandinavian J Immunol. (2019) 89:e12741. doi: 10.1111/sji.12741, PMID: 30549299

[6] Milano AF . Cancer of the larynx-20-year comparative survival and mortality analysis by age, sex, race, stage, grade, cohort entry time-period, disease duration and ICD-O-3 topographic primary sites-codes C32.0-9: A systematic review of 43,103 cases for diagnosis years 1975-2017: (NCI SEER*Stat 8.3.9). J insurance Med (New York NY). (2024) 51:92–110. doi: 10.17849/insm-51-2-92-110.1, PMID: 39266004

[7] Peng H He Y Hu Y Sheng S Maitiyasen M Li J . Berbamine promotes ferroptosis of esophageal squamous cell carcinoma by facilitating USP51-mediated GPX4 ubiquitination and degradation. Biomedicine pharmacotherapy. (2024) 179:117309. doi: 10.1016/j.biopha.2024.117309, PMID: 39151312

[8] Raggi C Taddei ML Rae C Braconi C Marra F . Metabolic reprogramming in cholangiocarcinoma. J Hepatol. (2022) 77:849–64. doi: 10.1016/j.jhep.2022.04.038, PMID: 35594992

[9] Szklener K Mazurek M Wieteska M Wacławska M Bilski M Mańdziuk S . New directions in the therapy of glioblastoma. Cancers. (2022) 14. doi: 10.3390/cancers14215377, PMID: 36358795 PMC9655599

[10] Gao Q Cheng X Gao X . Circ_0089761 accelerates colorectal cancer metastasis and immune escape via miR-27b-3p/PD-L1 axis. Physiol Rep. (2024) 12:e70137. doi: 10.14814/phy2.70137, PMID: 39632246 PMC11617067

[11] Özdemir S Arslan H . circRNA-based biomarker candidates for acute cypermethrin and chlorpyrifos toxication in the brain of Zebrafish (Danio rerio). Chemosphere. (2022) 298:134330. doi: 10.1016/j.chemosphere.2022.134330, PMID: 35304207

[12] Ren Y Manoharan T Liu B Cheng CZM En Siew B Cheong WK . Circular RNA as a source of neoantigens for cancer vaccines. J immunotherapy Cancer. (2024) 12. doi: 10.1136/jitc-2023-008402, PMID: 38508656 PMC10952939

[13] Xu C Jun E Okugawa Y Toiyama Y Borazanci E Bolton J . A circulating panel of circRNA biomarkers for the noninvasive and early detection of pancreatic ductal adenocarcinoma. Gastroenterology. (2024) 166:178–190.e116. doi: 10.1053/j.gastro.2023.09.050, PMID: 37839499 PMC10843014

[14] Bilotta MT Antignani A Fitzgerald DJ . Managing the TME to improve the efficacy of cancer therapy. Front Immunol. (2022) 13:954992. doi: 10.3389/fimmu.2022.954992, PMID: 36341428 PMC9630343

[15] Tiwari A Trivedi R Lin SY . Tumor microenvironment: barrier or opportunity towards effective cancer therapy. J Biomed Sci. (2022) 29:83. doi: 10.1186/s12929-022-00866-3, PMID: 36253762 PMC9575280

[16] White J White MPJ Wickremesekera A Peng L Gray C . The tumour microenvironment, treatment resistance and recurrence in glioblastoma. J Trans Med. (2024) 22:540. doi: 10.1186/s12967-024-05301-9, PMID: 38844944 PMC11155041

[17] Liu Y Wu Z Li Y Chen Y Zhao X Wu M . Metabolic reprogramming and interventions in angiogenesis. J advanced Res. (2025) 70:323–38. doi: 10.1016/j.jare.2024.05.001, PMID: 38704087 PMC11976431

[18] Xia L Oyang L Lin J Tan S Han Y Wu N . The cancer metabolic reprogramming and immune response. Mol Cancer. (2021) 20:28. doi: 10.1186/s12943-021-01316-8, PMID: 33546704 PMC7863491

[19] Yang K Wang X Song C He Z Wang R Xu Y . The role of lipid metabolic reprogramming in tumor microenvironment. Theranostics. (2023) 13:1774–808. doi: 10.7150/thno.82920, PMID: 37064872 PMC10091885

[20] Hernaez B Alcamí A . Poxvirus immune evasion. Annu Rev Immunol. (2024) 42:551–84. doi: 10.1146/annurev-immunol-090222-110227, PMID: 38941604

[21] Lima TS Lodoen MB . Mechanisms of human innate immune evasion by toxoplasma gondii. Front Cell infection Microbiol. (2019) 9:103. doi: 10.3389/fcimb.2019.00103, PMID: 31041194 PMC6476913

[22] Tauriello DVF Sancho E Batlle E . Overcoming TGFβ-mediated immune evasion in cancer. Nat Rev Cancer. (2022) 22:25–44. doi: 10.1038/s41568-021-00413-6, PMID: 34671117

[23] Peeraphatdit TB Wang J Odenwald MA Hu S Hart J Charlton MR . Hepatotoxicity from immune checkpoint inhibitors: A systematic review and management recommendation. Hepatol (Baltimore Md). (2020) 72:315–29. doi: 10.1002/hep.31227, PMID: 32167613

[24] Tan S Day D Nicholls SJ Segelov E . Immune checkpoint inhibitor therapy in oncology: current uses and future directions: JACC: cardioOncology state-of-the-art review. JACC CardioOncology. (2022) 4:579–97. doi: 10.1016/j.jaccao.2022.09.004, PMID: 36636451 PMC9830229

[25] Zhou P Gao Y Kong Z Wang J Si S Han W . Immune checkpoint inhibitors and acute kidney injury. Front Immunol. (2024) 15:1353339. doi: 10.3389/fimmu.2024.1353339, PMID: 38464524 PMC10920224

[26] Gao C Xu YJ Qi L Bao YF Zhang L Zheng L . CircRNA VIM silence synergizes with sevoflurane to inhibit immune escape and multiple oncogenic activities of esophageal cancer by simultaneously regulating miR-124/PD-L1 axis. Cell Biol Toxicol. (2022) 38:825–45. doi: 10.1007/s10565-021-09613-0, PMID: 34018092

[27] Wang B Liu W Zhang M Li Y Tang H Wang Y . Circ_0001947 encapsulated by small extracellular vesicles promotes gastric cancer progression and anti-PD-1 resistance by modulating CD8(+) T cell exhaustion. J nanobiotechnology. (2024) 22:563. doi: 10.1186/s12951-024-02826-5, PMID: 39272146 PMC11401313

[28] Yuan L Wang Y Cheng J Lin S Ma A Li K . Cancer-derived exosomal circTMEM56 enhances the efficacy of HCC radiotherapy through the miR-136-5p/STING axis. Cancer Biol Med. (2025) 22:396–411. doi: 10.20892/j.issn.2095-3941.2024.0544, PMID: 40269559 PMC12032838

[29] Fan L Xu G Zeng X . M2 macrophage-derived extracellular vesicles augment immune evasion and development of colorectal cancer via a circRNA_CCDC66/microRNA-342-3p/metadherin axis. Cytotechnology. (2023) 75:293–308. doi: 10.1007/s10616-023-00577-z, PMID: 37389129 PMC10299985

[30] Guo D Xu W Cui T Rong Q Wu Q . Protein-coding circular RNA enhances antiviral immunity via JAK/STAT pathway in Drosophila. mBio. (2024) 15:e0146924. doi: 10.1128/mbio.01469-24, PMID: 39158293 PMC11389369

[31] Li J Xu S Zhan Y Lv X Sun Z Man L . CircRUNX1 enhances the Warburg effect and immune evasion in non-small cell lung cancer through the miR-145/HK2 pathway. Cancer Lett. (2025) 620:217639. doi: 10.1016/j.canlet.2025.217639, PMID: 40090573

[32] Zhang LX Gao J Long X Zhang PF Yang X Zhu SQ . The circular RNA circHMGB2 drives immunosuppression and anti-PD-1 resistance in lung adenocarcinomas and squamous cell carcinomas via the miR-181a-5p/CARM1 axis. Mol Cancer. (2022) 21:110. doi: 10.1186/s12943-022-01586-w, PMID: 35525959 PMC9077876

[33] Miao Z Li J Wang Y Shi M Gu X Zhang X . Hsa_circ_0136666 stimulates gastric cancer progression and tumor immune escape by regulating the miR-375/PRKDC Axis and PD-L1 phosphorylation. Mol Cancer. (2023) 22:205. doi: 10.1186/s12943-023-01883-y, PMID: 38093288 PMC10718020

[34] Liu Z Wang T She Y Wu K Gu S Li L . N(6)-methyladenosine-modified circIGF2BP3 inhibits CD8(+) T-cell responses to facilitate tumor immune evasion by promoting the deubiquitination of PD-L1 in non-small cell lung cancer. Mol Cancer. (2021) 20:105. doi: 10.1186/s12943-021-01398-4, PMID: 34416901 PMC8377850

[35] Li Y Wang Z Gao P Cao D Dong R Zhu M . CircRHBDD1 promotes immune escape via IGF2BP2/PD-L1 signaling and acts as a nanotherapeutic target in gastric cancer. J Trans Med. (2024) 22:704. doi: 10.1186/s12967-024-05498-9, PMID: 39080693 PMC11289934

[36] Liu Z Zheng N Li J Li C Zheng D Jiang X . N6-methyladenosine-modified circular RNA QSOX1 promotes colorectal cancer resistance to anti-CTLA-4 therapy through induction of intratumoral regulatory T cells. Drug resistance updates: Rev commentaries antimicrobial Anticancer chemotherapy. (2022) 65:100886. doi: 10.1016/j.drup.2022.100886, PMID: 36370665

[37] Chen SW Zhu SQ Pei X Qiu BQ Xiong D Long X . Cancer cell-derived exosomal circUSP7 induces CD8(+) T cell dysfunction and anti-PD1 resistance by regulating the miR-934/SHP2 axis in NSCLC. Mol Cancer. (2021) 20:144. doi: 10.1186/s12943-021-01448-x, PMID: 34753486 PMC8576933

[38] Shi X Pang S Zhou J Yan G Gao R Wu H . Bladder-cancer-derived exosomal circRNA_0013936 promotes suppressive immunity by up-regulating fatty acid transporter protein 2 and down-regulating receptor-interacting protein kinase 3 in PMN-MDSCs. Mol Cancer. (2024) 23:52. doi: 10.1186/s12943-024-01968-2, PMID: 38461272 PMC10924381

[39] Zhuang M Zhang X Ji J Zhang H Shen L Zhu Y . Exosomal circ-0100519 promotes breast cancer progression via inducing M2 macrophage polarisation by USP7/NRF2 axis. Clin Trans Med. (2024) 14:e1763. doi: 10.1002/ctm2.1763, PMID: 39107958 PMC11303452

[40] Li K Lv J Wang J Wei Y Zhang Y Lin J . CircZNF609 inhibited bladder cancer immunotherapy sensitivity via enhancing fatty acid uptake through IGF2BP2/CD36 pathway. Int Immunopharmacol. (2024) 137:112485. doi: 10.1016/j.intimp.2024.112485, PMID: 38878487

[41] Li B Liang L Chen Y Liu J Wang Z Mao Y . Circ_0008287 promotes immune escape of gastric cancer cells through impairing microRNA-548c-3p-dependent inhibition of CLIC1. Int Immunopharmacol. (2022) 111:108918. doi: 10.1016/j.intimp.2022.108918, PMID: 35905561

[42] Jia L Wang Y Wang CY . circFAT1 promotes cancer stemness and immune evasion by promoting STAT3 activation. Advanced Sci (Weinheim Baden-Wurttemberg Germany). (2021) 8:2003376. doi: 10.1002/advs.202003376, PMID: 34258151 PMC8261519

[43] Wang Z Li Y Yang J Sun Y He Y Wang Y . CircCFL1 Promotes TNBC Stemness and Immunoescape via Deacetylation-Mediated c-Myc Deubiquitylation to Facilitate Mutant TP53 Transcription. Advanced Sci (Weinheim Baden-Wurttemberg Germany). (2024) 11:e2404628. doi: 10.1002/advs.202404628, PMID: 38981022 PMC11425638

[44] Hu Y Cai ZR Huang RZ Wang DS Ju HQ Chen DL . Circular RNA circPHLPP2 promotes tumor growth and anti-PD-1 resistance through binding ILF3 to regulate IL36γ transcription in colorectal cancer. Mol Cancer. (2024) 23:272. doi: 10.1186/s12943-024-02192-8, PMID: 39695693 PMC11658269

[45] Li J Dong X Kong X Wang Y Li Y Tong Y . Circular RNA hsa_circ_0067842 facilitates tumor metastasis and immune escape in breast cancer through HuR/CMTM6/PD-L1 axis. Biol direct. (2023) 18:48. doi: 10.1186/s13062-023-00397-3, PMID: 37592296 PMC10436663

[46] Zhang Z Huo W Li J . circATAD2 mitigates CD8(+) T cells antitumor immune surveillance in breast cancer via IGF2BP3/m(6)A/PD-L1 manner. Cancer immunology immunotherapy: CII. (2024) 73:130. doi: 10.1007/s00262-024-03705-6, PMID: 38748254 PMC11096152

[47] Xu YJ Zhao JM Gao C Ni XF Wang W Hu WW . Hsa_circ_0136666 activates Treg-mediated immune escape of colorectal cancer via miR-497/PD-L1 pathway. Cell signalling. (2021) 86:110095. doi: 10.1016/j.cellsig.2021.110095, PMID: 34320370

[48] Hong W Xue M Jiang J Zhang Y Gao X . Circular RNA circ-CPA4/let-7 miRNA/PD-L1 axis regulates cell growth, stemness, drug resistance and immune evasion in non-small cell lung cancer (NSCLC). J Exp Clin Cancer research: CR. (2020) 39:149. doi: 10.1186/s13046-020-01648-1, PMID: 32746878 PMC7397626

[49] Guo X Tan W Wang C . The emerging roles of exosomal circRNAs in diseases. Clin Trans Oncol. (2021) 23:1020–33. doi: 10.1007/s12094-020-02485-6, PMID: 32935262 PMC8084803

[50] Li C Ni YQ Xu H Xiang QY Zhao Y Zhan JK . Roles and mechanisms of exosomal non-coding RNAs in human health and diseases. Signal transduction targeted Ther. (2021) 6:383. doi: 10.1038/s41392-021-00779-x, PMID: 34753929 PMC8578673

[51] Xu Z Chen Y Ma L Chen Y Liu J Guo Y . Role of exosomal non-coding RNAs from tumor cells and tumor-associated macrophages in the tumor microenvironment. Mol Ther. (2022) 30:3133–54. doi: 10.1016/j.ymthe.2022.01.046, PMID: 35405312 PMC9552915

[52] Zhang F Jiang J Qian H Yan Y Xu W . Exosomal circRNA: emerging insights into cancer progression and clinical application potential. J Hematol Oncol. (2023) 16:67. doi: 10.1186/s13045-023-01452-2, PMID: 37365670 PMC10294326

[53] Can C Yang X Jia H Wu H Guo X Wei Y . Exosomal circ_0006896 promotes AML progression via interaction with HDAC1 and restriction of antitumor immunity. Mol Cancer. (2025) 24:4. doi: 10.1186/s12943-024-02203-8, PMID: 39762891 PMC11702195

[54] Fu J Liu F Bai S Jiang X Song H Zhang M . Circular RNA CDYL facilitates hepatocellular carcinoma stemness and PD-L1(+) exosomes-mediated immunotherapy resistance via stabilizing hornerin protein by blocking synoviolin 1-mediated ubiquitination. Int J Biol macromolecules. (2025) 310:143246. doi: 10.1016/j.ijbiomac.2025.143246, PMID: 40250664

[55] Cai ZR Hu Y Liao K Li H Chen DL Ju HQ . Circular RNAs: Emerging regulators of glucose metabolism in cancer. Cancer Lett. (2023) 552:215978. doi: 10.1016/j.canlet.2022.215978, PMID: 36283584

[56] Geng Y Wang M Wu Z Jia J Yang T Yu L . Research progress of circRNA in Malignant tumour metabolic reprogramming. RNA Biol. (2023) 20:641–51. doi: 10.1080/15476286.2023.2247877, PMID: 37599427 PMC10443989

[57] Liu S Jiao B Zhao H Liang X Jin F Liu X . LncRNAs-circRNAs as rising epigenetic binary superstars in regulating lipid metabolic reprogramming of cancers. Advanced Sci (Weinheim Baden-Wurttemberg Germany). (2024) 11:e2303570. doi: 10.1002/advs.202303570, PMID: 37939296 PMC10767464

[58] Safi A Saberiyan M Sanaei MJ Adelian S Davarani Asl F Zeinaly M . The role of noncoding RNAs in metabolic reprogramming of cancer cells. Cell Mol Biol Lett. (2023) 28:37. doi: 10.1186/s11658-023-00447-8, PMID: 37161350 PMC10169341

[59] Zhang B Yang L He Y Han D Qi P Shang P . Role and mechanisms of noncoding RNAs in the regulation of metabolic reprogramming in bladder cancer (Review). Int J Mol Med. (2023) 52. doi: 10.3892/ijmm.2023.5282, PMID: 37477143

[60] Zhang Y Mao Q Xia Q Cheng J Huang Z Li Y . Noncoding RNAs link metabolic reprogramming to immune microenvironment in cancers. J Hematol Oncol. (2021) 14:169. doi: 10.1186/s13045-021-01179-y, PMID: 34654454 PMC8518176

[61] Li P Ren X Zheng Y Sun J Ye G . Tumor promoting effect of circ_002172 associates with induced immune escape in breast cancer via the miR-296-5p/CXCL12 axis. Int Immunopharmacol. (2022) 106:108530. doi: 10.1016/j.intimp.2022.108530, PMID: 35240495

[62] Zhang R Shang L Nan J Niu K Dai J Jin X . Circ-METTL15 contributes to the proliferation, metastasis, immune escape and restrains apoptosis in lung cancer by regulating miR-1299/PDL1 axis. Autoimmunity. (2022) 55:8–20. doi: 10.1080/08916934.2021.2001801, PMID: 34796777

[63] Feng Z Meng S Zhou H Xu Z Tang Y Li P . Functions and potential applications of circular RNAs in cancer stem cells. Front Oncol. (2019) 9:500. doi: 10.3389/fonc.2019.00500, PMID: 31263676 PMC6584801

[64] Hui Y Wenguang Y Wei S Haoran W Shanglei N Ju L . circSLC4A7 accelerates stemness and progression of gastric cancer by interacting with HSP90 to activate NOTCH1 signaling pathway. Cell Death Dis. (2023) 14:452. doi: 10.1038/s41419-023-05976-w, PMID: 37474578 PMC10359325

[65] Kyriazi AA Papiris E Kitsos Kalyvianakis K Sakellaris G Baritaki S . Dual effects of non-coding RNAs (ncRNAs) in cancer stem cell biology. Int J Mol Sci. (2020) 21. doi: 10.3390/ijms21186658, PMID: 32932969 PMC7556003

[66] Wang JB Lin TX Fan DH Gao YX Chen YJ Wu YK . CircUBA2 promotes the cancer stem cell-like properties of gastric cancer through upregulating STC1 via sponging miR-144-5p. Cancer Cell Int. (2024) 24:276. doi: 10.1186/s12935-024-03423-0, PMID: 39103836 PMC11302268

[67] Kharkar PS . Cancer stem cell (CSC) inhibitors: a review of recent patents (2012-2015). Expert Opin Ther patents. (2017) 27:753–61. doi: 10.1080/13543776.2017.1325465, PMID: 28460551

[68] Najafi M Mortezaee K Majidpoor J . Cancer stem cell (CSC) resistance drivers. Life Sci. (2019) 234:116781. doi: 10.1016/j.lfs.2019.116781, PMID: 31430455

[69] Nassar D Blanpain C . Cancer stem cells: basic concepts and therapeutic implications. Annu Rev Pathol. (2016) 11:47–76. doi: 10.1146/annurev-pathol-012615-044438, PMID: 27193450

[70] Chen H Li Y . Circular RNA hsa_circ_0000915 promotes propranolol resistance of hemangioma stem cells in infantile haemangiomas. Hum Genomics. (2022) 16:43. doi: 10.1186/s40246-022-00416-w, PMID: 36167680 PMC9513930

[71] Fei Y Cao D Li Y Wang Z Dong R Zhu M . Circ_0008315 promotes tumorigenesis and cisplatin resistance and acts as a nanotherapeutic target in gastric cancer. J nanobiotechnology. (2024) 22:519. doi: 10.1186/s12951-024-02760-6, PMID: 39210348 PMC11360491

[72] Hui B Zhou C Xu Y Wang R Dong Y Zhou Y . Exosomes secreted by Fusobacterium nucleatum-infected colon cancer cells transmit resistance to oxaliplatin and 5-FU by delivering hsa_circ_0004085. J nanobiotechnology. (2024) 22:62. doi: 10.1186/s12951-024-02331-9, PMID: 38360615 PMC10867993

[73] Tang YF Liu ZH Zhang LY Shi SH Xu S Ma JA . circ_PPAPDC1A promotes Osimertinib resistance by sponging the miR-30a-3p/IGF1R pathway in non-small cell lung cancer (NSCLC). Mol Cancer. (2024) 23:91. doi: 10.1186/s12943-024-01998-w, PMID: 38715012 PMC11075361

[74] Wu S Lv X Wei H Chen W Zheng J Li X . Circ-ILF2 in oral squamous cell carcinoma promotes cisplatin resistance and induces M2 polarization of macrophages. J Cell Mol Med. (2023) 27:4133–44. doi: 10.1111/jcmm.17998, PMID: 37864310 PMC10746935

[75] Xu L Ma X Zhang X Zhang C Zhang Y Gong S . hsa_circ_0007919 induces LIG1 transcription by binding to FOXA1/TET1 to enhance the DNA damage response and promote gemcitabine resistance in pancreatic ductal adenocarcinoma. Mol Cancer. (2023) 22:195. doi: 10.1186/s12943-023-01887-8, PMID: 38044421 PMC10694898

[76] Wang Y Cui Y Li X Jin SH Wang H Gaipl US . CircRNAs: functions and emerging roles in cancer and immunotherapy. BMC Med. (2025) 23:477. doi: 10.1186/s12916-025-04306-5, PMID: 40817061 PMC12357350

[77] Huang S Xu J Baran N Ma W . Advancing the next generation of cancer treatment with circular RNAs in CAR-T cell therapy. Biomedicine pharmacotherapy = Biomedecine pharmacotherapie. (2024) 181:117753. doi: 10.1016/j.biopha.2024.117753, PMID: 39667221

[78] Wang X Ma J Dang Y Lei F . Circular RNAs and immunotherapy in retinoblastoma: emerging biomarkers and precision therapeutic strategies. Front Immunol. (2025) 16:1666606. doi: 10.3389/fimmu.2025.1666606, PMID: 41041300 PMC12484201

[79] Cai J Liu Z Chen S Zhang J Li H Wang X . Engineered circular RNA-based DLL3-targeted CAR-T therapy for small cell lung cancer. Exp Hematol Oncol. (2025) 14:35. doi: 10.1186/s40164-025-00625-8, PMID: 40075480 PMC11905684

[80] Su PL Chakravarthy K Furuya N Brownstein J Yu J Long M . DLL3-guided therapies in small-cell lung cancer: from antibody-drug conjugate to precision immunotherapy and radioimmunotherapy. Mol Cancer. (2024) 23:97. doi: 10.1186/s12943-024-02012-z, PMID: 38730427 PMC11084107

[81] Tang Y Jiang M Jiang HM Ye ZJ Huang YS Li XS . The roles of circRNAs in liver cancer immunity. Front Oncol. (2020) 10:598464. doi: 10.3389/fonc.2020.598464, PMID: 33614486 PMC7890029

[82] Wei S Hu W Feng J Geng Y . Promotion or remission: a role of noncoding RNAs in colorectal cancer resistance to anti-EGFR therapy. Cell communication signaling: CCS. (2022) 20:150. doi: 10.1186/s12964-022-00960-x, PMID: 36131281 PMC9490904

[83] Zhou C Li W Liang Z Wu X Cheng S Peng J . Mutant KRAS-activated circATXN7 fosters tumor immunoescape by sensitizing tumor-specific T cells to activation-induced cell death. Nat Commun. (2024) 15:499. doi: 10.1038/s41467-024-44779-1, PMID: 38216551 PMC10786880

[84] Li B Zhu L Lu C Wang C Wang H Jin H . circNDUFB2 inhibits non-small cell lung cancer progression via destabilizing IGF2BPs and activating anti-tumor immunity. Nat Commun. (2021) 12:295. doi: 10.1038/s41467-020-20527-z, PMID: 33436560 PMC7804955

[85] Firoozi Z Mohammadisoleimani E Shahi A Naghizadeh MM Mirzaei E Asad AG . Hsa_circ_0000479/Hsa-miR-149-5p/RIG-I, IL-6 axis: A potential novel pathway to regulate immune response against COVID-19. Can J Infect Dis Med Microbiol. (2022) 2022:2762582. doi: 10.1155/2022/2762582, PMID: 36081604 PMC9448594

[86] Mohammadisoleimani E Firoozi Z Naghizadeh MM Ghanbari Asad A Pezeshki B Gholampour Y . Upregulation of hsa_circ_0004812 promotes COVID-19 cytokine storm via hsa-miR-1287-5p/IL6R, RIG-I axis. J Clin Lab Anal. (2022) 36:e24666. doi: 10.1002/jcla.24666, PMID: 35989496 PMC9538103

[87] Wang W Ma Z Feng X Ren J Sun S Shao Y . TfR1 mediated iron metabolism dysfunction as a potential therapeutic target for osteoarthritis. Arthritis Res Ther. (2024) 26:71. doi: 10.1186/s13075-024-03304-x, PMID: 38493104 PMC10943767

[88] Ahmed A Tait SWG . Targeting immunogenic cell death in cancer. Mol Oncol. (2020) 14:2994–3006. doi: 10.1002/1878-0261.12851, PMID: 33179413 PMC7718954

[89] Bains SN Nash P Fonacier L . Irritant contact dermatitis. Clin Rev Allergy Immunol. (2019) 56:99–109. doi: 10.1007/s12016-018-8713-0, PMID: 30293200

[90] Kroemer G Galluzzi L Kepp O Zitvogel L . Immunogenic cell death in cancer therapy. Annu Rev Immunol. (2013) 31:51–72. doi: 10.1146/annurev-immunol-032712-100008, PMID: 23157435

[91] Zhang Y Song X Feng Y Qian Y Chen B Zhang T . The circRNA cEMSY Induces Immunogenic Cell Death and Boosts Immunotherapy Efficacy in Lung Adenocarcinoma. Cancer Res. (2025) 85:497–514. doi: 10.1158/0008-5472.CAN-24-1484, PMID: 39531509 PMC11786956

[92] Chen L Wang C Sun H Wang J Liang Y Wang Y . The bioinformatics toolbox for circRNA discovery and analysis. Briefings Bioinf. (2021) 22:1706–28. doi: 10.1093/bib/bbaa001, PMID: 32103237 PMC7986655

[93] Chen XL Tan QD Chen KJ Zheng DN Deng HW He S . CircRNA and stroke: new insight of potential biomarkers and therapeutic targets. Neurochemical Res. (2024) 49:557–67. doi: 10.1007/s11064-023-04077-6, PMID: 38063946

[94] Costa MC Calderon-Dominguez M Mangas A Campuzano O Sarquella-Brugada G Ramos M . Circulating circRNA as biomarkers for dilated cardiomyopathy etiology. J Mol Med (Berlin Germany). (2021) 99:1711–25. doi: 10.1007/s00109-021-02119-6, PMID: 34498126 PMC8599237

[95] Zhang Y Luo J Yang W Ye WC . CircRNAs in colorectal cancer: potential biomarkers and therapeutic targets. Cell Death Dis. (2023) 14:353. doi: 10.1038/s41419-023-05881-2, PMID: 37296107 PMC10250185

[96] Zhang Y Tao K Ding L Zhao Y . Assessing biomarkers for post-surgical wound healing: A meta-analysis of exosome-based CircRNA in breast cancer recovery. Int Wound J. (2024) 21:e14723. doi: 10.1111/iwj.14723, PMID: 38379248 PMC10830351

[97] Li H Lin R Zhang Y Zhu Y Huang S Lan J . N6-methyladenosine-modified circPLPP4 sustains cisplatin resistance in ovarian cancer cells via PIK3R1 upregulation. Mol Cancer. (2024) 23:5. doi: 10.1186/s12943-023-01917-5, PMID: 38184597 PMC10770956

[98] Liu Y Yue J Jiang Y Tian X Shu A . The role of circRNA in insulin resistance and its progression induced by adipose inflammation. J Diabetes its complications. (2025) 39:109042. doi: 10.1016/j.jdiacomp.2025.109042, PMID: 40279985

[99] Wang T He M Zhang X Guo Z Wang P Long F . Deciphering the impact of circRNA-mediated autophagy on tumor therapeutic resistance: a novel perspective. Cell Mol Biol Lett. (2024) 29:60. doi: 10.1186/s11658-024-00571-z, PMID: 38671354 PMC11046940

[100] Zhang J Yu Q Zhu W Sun X . Recent advances in the role of circRNA in cisplatin resistance in tumors. Cancer Gene Ther. (2025) 32:497–506. doi: 10.1038/s41417-025-00899-4, PMID: 40148680

[101] Wu R Yu S Bi A Li Y Tiek D Yu K . Therapeutic targeting of circTNK2 with nanoparticles restores tamoxifen sensitivity and enhances NK cell-mediated immunity in ER-positive breast cancer. Cancer Lett. (2025) 627:217823. doi: 10.1016/j.canlet.2025.217823, PMID: 40419081

[102] Lin H Zhu S Chen Y Lu J Xie C Liao C . Targeting cTRIP12 counteracts ferroptosis resistance and augments sensitivity to immunotherapy in pancreatic cancer. Drug resistance updates. (2025) 81:101240. doi: 10.1016/j.drup.2025.101240, PMID: 40154160

[103] Guan L Hao Q Shi F Gao B Wang M Zhou X . Regulation of the tumor immune microenvironment by cancer-derived circular RNAs. Cell Death Dis. (2023) 14:132. doi: 10.1038/s41419-023-05647-w, PMID: 36797245 PMC9935907

[104] Liu T Long K Zhu Z Song Y Chen C Xu G . Roles of circRNAs in regulating the tumor microenvironment. Med Oncol (Northwood London England). (2023) 40:329. doi: 10.1007/s12032-023-02194-4, PMID: 37819576 PMC10567871

[105] Xu HZ Lin XY Xu YX Xue HB Lin S Xu TW . An emerging research: the role of hepatocellular carcinoma-derived exosomal circRNAs in the immune microenvironment. Front Immunol. (2023) 14:1227150. doi: 10.3389/fimmu.2023.1227150, PMID: 37753074 PMC10518420

[106] Zhang Q Wang W Zhou Q Chen C Yuan W Liu J . Roles of circRNAs in the tumour microenvironment. Mol Cancer. (2020) 19:14. doi: 10.1186/s12943-019-1125-9, PMID: 31973726 PMC6977266

[107] Xu BB Huang Y Zheng ED Wang JY Zhang CJ Geng XG . Hsa_circ_0072309 is a prognostic biomarker and is correlated with immune infiltration in gastric cancer. Heliyon. (2023) 9:e13191. doi: 10.1016/j.heliyon.2023.e13191, PMID: 36852074 PMC9958299

[108] Zang J Xiao L Shi X Liu S Wang Y Sun B . Hsa_circ_0001479 accelerates tumorigenesis of gastric cancer and mediates immune escape. Int Immunopharmacol. (2023) 124:110887. doi: 10.1016/j.intimp.2023.110887, PMID: 37683398

[109] Wu ZH Li ZW Yang DL Liu J . Development and validation of a pyroptosis-related long non-coding RNA signature for hepatocellular carcinoma. Front Cell Dev Biol. (2021) 9:713925. doi: 10.3389/fcell.2021.713925, PMID: 34869306 PMC8634266

[110] Lin Z Ji Y Zhou J Li G Wu Y Liu W . Exosomal circRNAs in cancer: Implications for therapy resistance and biomarkers. Cancer Lett. (2023) 566:216245. doi: 10.1016/j.canlet.2023.216245, PMID: 37247772

[111] Liu Q Li S . Exosomal circRNAs: Novel biomarkers and therapeutic targets for urinary tumors. Cancer Lett. (2024) 588:216759. doi: 10.1016/j.canlet.2024.216759, PMID: 38417667

[112] Ma Y Liu Y Jiang Z . CircRNAs: A new perspective of biomarkers in the nervous system. Biomedicine pharmacotherapy = Biomedecine pharmacotherapie. (2020) 128:110251. doi: 10.1016/j.biopha.2020.110251, PMID: 32480219

[113] Verduci L Tarcitano E Strano S Yarden Y Blandino G . CircRNAs: role in human diseases and potential use as biomarkers. Cell Death Dis. (2021) 12:468. doi: 10.1038/s41419-021-03743-3, PMID: 33976116 PMC8113373

[114] Wang D Li R Jiang J Qian H Xu W . Exosomal circRNAs: Novel biomarkers and therapeutic targets for gastrointestinal tumors. Biomedicine pharmacotherapy = Biomedecine pharmacotherapie. (2023) 157:114053. doi: 10.1016/j.biopha.2022.114053, PMID: 36462315

[115] Wang L Lu M Li W Fan R Wen S Xiao W . Significance of circRNAs as biomarkers for systemic lupus erythematosus: a systematic review and meta-analysis. J Int Med Res. (2022) 50:3000605221103546. doi: 10.1177/03000605221103546, PMID: 35796516 PMC9274425

[116] Cui J Wang J Liu L Zou C Zhao Y Xue Z . Presence and prospects of exosomal circRNAs in cancer (Review). Int J Oncol. (2023) 62. doi: 10.3892/ijo.2023.5495, PMID: 36866755

[117] Xu Y Han J Zhang X Zhang X Song J Gao Z . Exosomal circRNAs in gastrointestinal cancer: Role in occurrence, development, diagnosis and clinical application (Review). Oncol Rep. (2024) 51. doi: 10.3892/or.2023.8678, PMID: 38099408 PMC10777447

[118] Zhao J Yan W Huang W Li Y . Circ_0010235 facilitates lung cancer development and immune escape by regulating miR-636/PDL1 axis. Thorac Cancer. (2022) 13:965–76. doi: 10.1111/1759-7714.14338, PMID: 35167195 PMC8977160

[119] Jiang X Liu B Nie Z Duan L Xiong Q Jin Z . The role of m6A modification in the biological functions and diseases. Signal transduction targeted Ther. (2021) 6:74. doi: 10.1038/s41392-020-00450-x, PMID: 33611339 PMC7897327

[120] Barilani M Peli V Manzini P Pistoni C Rusconi F Pinatel EM . Extracellular vesicles from human induced pluripotent stem cells exhibit a unique microRNA and circRNA signature. Int J Biol Sci. (2024) 20:6255–78. doi: 10.7150/ijbs.100113, PMID: 39664576 PMC11628337

[121] Diao X Guo C Zheng H Zhao K Luo Y An M . SUMOylation-triggered ALIX activation modulates extracellular vesicles circTLCD4-RWDD3 to promote lymphatic metastasis of non-small cell lung cancer. Signal transduction targeted Ther. (2023) 8:426. doi: 10.1038/s41392-023-01685-0, PMID: 37925421 PMC10625632

[122] Du X Hu W Li X Gao Y Li J Hu X . CircRNA-miRNA-mRNA networks in plasma extracellular vesicles as biomarkers for first-onset schizophrenia. BMC Psychiatry. (2025) 25:688. doi: 10.1186/s12888-025-07073-y, PMID: 40634861 PMC12239366

[123] Huang Q Chu Z Wang Z Li Q Meng S Lu Y . circCDK13-loaded small extracellular vesicles accelerate healing in preclinical diabetic wound models. Nat Commun. (2024) 15:3904. doi: 10.1038/s41467-024-48284-3, PMID: 38724502 PMC11082226

[124] Yang L Han B Zhang Z Wang S Bai Y Zhang Y . Extracellular vesicle-Mediated delivery of circular RNA SCMH1 promotes functional recovery in rodent and nonhuman primate ischemic stroke models. Circulation. (2020) 142:556–74. doi: 10.1161/CIRCULATIONAHA.120.045765, PMID: 32441115

[125] Yao Y Chen C Wang J Xuan H Chen X Li Z . Circular RNA circATP9A promotes non-small cell lung cancer progression by interacting with HuR and by promoting extracellular vesicles-mediated macrophage M2 polarization. J Exp Clin Cancer research: CR. (2023) 42:330. doi: 10.1186/s13046-023-02916-6, PMID: 38049814 PMC10696866

[126] Zhou J Song Q Li H Han Y Pu Y Li L . Targeting circ-0034880-enriched tumor extracellular vesicles to impede SPP1(high)CD206(+) pro-tumor macrophages mediated pre-metastatic niche formation in colorectal cancer liver metastasis. Mol Cancer. (2024) 23:168. doi: 10.1186/s12943-024-02086-9, PMID: 39164758 PMC11334400

[127] Li R Tian X Jiang J Qian H Shen H Xu W . CircRNA CDR1as: a novel diagnostic and prognostic biomarker for gastric cancer. Biomarkers: Biochem Indic exposure response susceptibility to chemicals. (2023) 28:448–57. doi: 10.1080/1354750X.2023.2206984, PMID: 37128800

[128] Wang L Xiao S Zheng Y Gao Z . CircRNA circSLIT2 is a novel diagnostic and prognostic biomarker for gastric cancer. Wiener klinische Wochenschrift. (2023) 135:472–7. doi: 10.1007/s00508-023-02155-x, PMID: 37074418

[129] Yao G Niu W Zhu X He M Kong L Chen S . hsa_circRNA_104597: a novel potential diagnostic and therapeutic biomarker for schizophrenia. Biomarkers Med. (2019) 13:331–40. doi: 10.2217/bmm-2018-0447, PMID: 30781971

[130] Das A Das D Panda AC . Quantification of circular RNAs using digital droplet PCR. J visualized experiments: JoVE. (2022) (187). doi: 10.3791/64464-v, PMID: 36190278 PMC7614906

[131] Li T Shao Y Fu L Xie Y Zhu L Sun W . Plasma circular RNA profiling of patients with gastric cancer and their droplet digital RT-PCR detection. J Mol Med (Berlin Germany). (2018) 96:85–96. doi: 10.1007/s00109-017-1600-y, PMID: 29098316

[132] Masante L Susin G Baudet ML . Droplet digital PCR for the detection and quantification of bona fide circRNAs. Methods Mol Biol (Clifton NJ). (2024) 2765:107–26. doi: 10.1007/978-1-0716-3678-7_6, PMID: 38381336

[133] Peng J Li F Xu X Hu S . Single-Cell Analysis of circRNA Using ddPCR. Methods Mol Biol (Clifton NJ). (2023) 2689:169–77. doi: 10.1007/978-1-0716-3323-6_13, PMID: 37430054

[134] Wang K Yin H Li S Wan Y Xiao M Yuan X . Quantitative detection of circular RNA and microRNA at point-of-care using droplet digital CRISPR/Cas13a platform. Biosensors bioelectronics. (2025) 267:116825. doi: 10.1016/j.bios.2024.116825, PMID: 39369515

[135] Chen C Yu H Han F Lai X Ye K Lei S . Tumor-suppressive circRHOBTB3 is excreted out of cells via exosome to sustain colorectal cancer cell fitness. Mol Cancer. (2022) 21:46. doi: 10.1186/s12943-022-01511-1, PMID: 35148775 PMC8832727

[136] Kim T Yum K Lee JB . Therapeutic potential of circular antisense oligonucleotides in gene silencing and RNA condensate degradation. Int J Biol macromolecules. (2025) 322:146970. doi: 10.1016/j.ijbiomac.2025.146970, PMID: 40840752

[137] Stulz R Lerche M Luige O Taylor A Geschwindner S Ghidini A . An enhanced biophysical screening strategy to investigate the affinity of ASOs for their target RNA. RSC Chem Biol. (2023) 4:1123–30. doi: 10.1039/D3CB00072A, PMID: 38033730 PMC10685824

[138] Titze-de-Almeida SS Titze-de-Almeida R . Progress in circRNA-targeted therapy in experimental parkinson’s disease. Pharmaceutics. (2023) 15. doi: 10.3390/pharmaceutics15082035, PMID: 37631249 PMC10459713

[139] Tuo B Chen Z Dang Q Chen C Zhang H Hu S . Roles of exosomal circRNAs in tumour immunity and cancer progression. Cell Death Dis. (2022) 13:539. doi: 10.1038/s41419-022-04949-9, PMID: 35676257 PMC9177590

[140] Wu X Shi M Lian Y Zhang H . Exosomal circRNAs as promising liquid biopsy biomarkers for glioma. Front Immunol. (2023) 14:1039084. doi: 10.3389/fimmu.2023.1039084, PMID: 37122733 PMC10140329

[141] Zhang XP Pei JP Zhang CD Yusupu M Han MH Dai DQ . Exosomal circRNAs: A key factor of tumor angiogenesis and therapeutic intervention. Biomedicine pharmacotherapy = Biomedecine pharmacotherapie. (2022) 156:113921. doi: 10.1016/j.biopha.2022.113921, PMID: 36411614

[142] Patni H Chaudhary R Kumar A . Unleashing nanotechnology to redefine tumor-associated macrophage dynamics and non-coding RNA crosstalk in breast cancer. Nanoscale. (2024) 16:18274–94. doi: 10.1039/D4NR02795G, PMID: 39292162

[143] Meng H Li R Xie Y Mo Z Zhai H Zhang G . Nanoparticles Mediated circROBO1 Silencing to Inhibit Hepatocellular Carcinoma Progression by Modulating miR-130a-5p/CCNT2 Axis. Int J nanomedicine. (2023) 18:1677–93. doi: 10.2147/IJN.S399318, PMID: 37020690 PMC10069521

[144] Wu S Huang J Li Y Liu Z Zhao L . Integrated Analysis of lncRNA and circRNA Mediated ceRNA Regulatory Networks in Skin Reveals Innate Immunity Differences Between Wild-Type and Yellow Mutant Rainbow Trout (Oncorhynchus mykiss). Front Immunol. (2022) 13:802731. doi: 10.3389/fimmu.2022.802731, PMID: 35655786 PMC9152293

[145] Chen YG Kim MV Chen X Batista PJ Aoyama S Wilusz JE . Sensing self and foreign circular RNAs by intron identity. Mol Cell. (2017) 67:228–238.e225. doi: 10.1016/j.molcel.2017.05.022, PMID: 28625551 PMC5610545

[146] Qin L Lin J Xie X . CircRNA-9119 suppresses poly I:C induced inflammation in Leydig and Sertoli cells via TLR3 and RIG-I signal pathways. Mol Med (Cambridge Mass). (2019) 25:28. doi: 10.1186/s10020-019-0094-1, PMID: 31195953 PMC6567632

[147] Song J Zhao W Zhang X Tian W Zhao X Ma L . Mutant RIG-I enhances cancer-related inflammation through activation of circRIG-I signaling. Nat Commun. (2022) 13:7096. doi: 10.1038/s41467-022-34885-3, PMID: 36402769 PMC9675819

[148] Mu M Niu W Chu F Dong Q Hu S Niu C . CircSOBP suppresses the progression of glioma by disrupting glycolysis and promoting the MDA5-mediated immune response. iScience. (2023) 26:107897. doi: 10.1016/j.isci.2023.107897, PMID: 37766977 PMC10520879

[149] Zhang C Zhang C Ji J Xiong X Lu Y . Hsa_circ_0012919 regulates expression of MDA5 by miR-125a-3p in CD4+ T cells of systemic lupus erythematous. Lupus. (2020) 29:727–34. doi: 10.1177/0961203320920706, PMID: 32321346

[150] Della Bella E Koch J Baerenfaller K . Translation and emerging functions of non-coding RNAs in inflammation and immunity. Allergy. (2022) 77:2025–37. doi: 10.1111/all.15234, PMID: 35094406 PMC9302665

[151] Tornesello AL Cerasuolo A Starita N Amiranda S Cimmino TP . Bonelli P et al: Emerging role of endogenous peptides encoded by non-coding RNAs in cancer biology. Non-coding RNA Res. (2025) 10:231–41. doi: 10.1016/j.ncrna.2024.10.006, PMID: 39554691 PMC11567935

[152] Wen K Chen X Gu J Chen Z Wang Z . Beyond traditional translation: ncRNA derived peptides as modulators of tumor behaviors. J Biomed Sci. (2024) 31:63. doi: 10.1186/s12929-024-01047-0, PMID: 38877495 PMC11177406

[153] Iyer S Mir A Vega-Badillo J Roscoe BP Ibraheim R Zhu LJ . Efficient homology-Directed repair with circular single-Stranded DNA donors. CRISPR J. (2022) 5:685–701. doi: 10.1089/crispr.2022.0058, PMID: 36070530 PMC9595650

[154] Qian H Maghsoudloo M Kaboli PJ Babaeizad A Cui Y Fu J . Decoding the Promise and Challenges of miRNA-Based Cancer Therapies: An Essential Update on miR-21, miR-34, and miR-155. Int J Med Sci. (2024) 21:2781–98. doi: 10.7150/ijms.102123, PMID: 39512697 PMC11539376

[155] Triska J Mathew C Zhao Y Chen YE Birnbaum Y . Circular RNA as therapeutic targets in atherosclerosis: are we running in circles? J Clin Med. (2023) 12., PMID: 37445481 10.3390/jcm12134446 PMC10342353

[156] Zhang L Liang D Chen C Wang Y Amu G Yang J . Circular siRNAs for reducing off-Target effects and enhancing long-Term gene silencing in cells and mice. Mol Ther Nucleic Acids. (2018) 10:237–44. doi: 10.1016/j.omtn.2017.12.007, PMID: 29499936 PMC5768153

[157] Zhang YW Li S Wang SM Li XQ Cui MR Kang B . An intelligent DNA nanomachine for amplified MicroRNA imaging and MicroRNA-Guided efficient gene silencing. Talanta. (2023) 265:124820. doi: 10.1016/j.talanta.2023.124820, PMID: 37331040

[158] Alshehry Y Liu X Zhang Y Zhu G . Investigation of the impact of lipid nanoparticle compositions on the delivery and T cell response of circRNA vaccine. J Controlled release. (2025) 381:113617. doi: 10.1016/j.jconrel.2025.113617, PMID: 40107513 PMC11994274

[159] Feng Z Zhang X Zhou J Li Q Chu L Di G . An *in vitro*-transcribed circular RNA targets the mitochondrial inner membrane cardiolipin to ablate EIF4G2(+)/PTBP1(+) pan-adenocarcinoma. Nat Cancer. (2024) 5:30–46. doi: 10.1038/s43018-023-00650-8, PMID: 37845485

[160] Wan J Wang Z Wang L Wu L Zhang C Zhou M . Circular RNA vaccines with long-term lymph node-targeting delivery stability after lyophilization induce potent and persistent immune responses. mBio. (2024) 15:e0177523. doi: 10.1128/mbio.01775-23, PMID: 38078742 PMC10790773

[161] Wu Z Zuo X Zhang W Li Y Gui R Leng J . m6A-Modified circTET2 Interacting with HNRNPC Regulates Fatty Acid Oxidation to Promote the Proliferation of Chronic Lymphocytic Leukemia. Advanced Sci (Weinheim Baden-Wurttemberg Germany). (2023) 10:e2304895. doi: 10.1002/advs.202304895, PMID: 37821382 PMC10700176

[162] Digby B Finn S Broin PÓ . Computational approaches and challenges in the analysis of circRNA data. BMC Genomics. (2024) 25:527. doi: 10.1186/s12864-024-10420-0, PMID: 38807085 PMC11134749

[163] Dong J Zeng Z Huang Y Chen C Cheng Z Zhu Q . Challenges and opportunities for circRNA identification and delivery. Crit Rev Biochem Mol Biol. (2023) 58:19–35. doi: 10.1080/10409238.2023.2185764, PMID: 36916323

[164] Li D Guo J Ni X Sun G Bao H . The progress and challenges of circRNA for diabetic foot ulcers: A mini-review. Front Endocrinol. (2022) 13:1019935. doi: 10.3389/fendo.2022.1019935, PMID: 36531481 PMC9747764

[165] Li X Yang L Chen LL . The biogenesis, functions, and challenges of circular RNAs. Mol Cell. (2018) 71:428–42. doi: 10.1016/j.molcel.2018.06.034, PMID: 30057200

[166] Jarlstad Olesen MT Kristensen LS . Circular RNAs as microRNA sponges: evidence and controversies. Essays Biochem. (2021) 65:685–96. doi: 10.1042/EBC20200060, PMID: 34028529

[167] Kristensen LS Andersen MS Stagsted LVW Ebbesen KK Hansen TB Kjems J . The biogenesis, biology and characterization of circular RNAs. Nat Rev Genet. (2019) 20:675–91. doi: 10.1038/s41576-019-0158-7, PMID: 31395983

[168] Mafi A Yadegar N Salami M Salami R Vakili O Aghadavod E . Circular RNAs; powerful microRNA sponges to overcome diabetic nephropathy. Pathology Res Pract. (2021) 227:153618. doi: 10.1016/j.prp.2021.153618, PMID: 34649056

[169] Zhang G Zhu Y Jin C Shi Q An X Song L . CircRNA_0078767 promotes osteosarcoma progression by increasing CDK14 expression through sponging microRNA-330-3p. Chemico-biological Interact. (2022) 360:109903. doi: 10.1016/j.cbi.2022.109903, PMID: 35307379

[170] Zhang S Wang X Chen G Tong L Dai T Wang L . CircRNA Galntl6 sponges miR-335 to ameliorate stress-induced hypertension through upregulating Lig3 in rostral ventrolateral medulla. Redox Biol. (2023) 64:102782. doi: 10.1016/j.redox.2023.102782, PMID: 37315345 PMC10363431

[171] Zhang ZH Wang Y Zhang Y Zheng SF Feng T Tian X . The function and mechanisms of action of circular RNAs in Urologic Cancer. Mol Cancer. (2023) 22:61. doi: 10.1186/s12943-023-01766-2, PMID: 36966306 PMC10039696

[172] Du WW Zhang C Yang W Yong T Awan FM Yang BB . Identifying and characterizing circRNA-protein interaction. Theranostics. (2017) 7:4183–91. doi: 10.7150/thno.21299, PMID: 29158818 PMC5695005

[173] Huang A Zheng H Wu Z Chen M Huang Y . Circular RNA-protein interactions: functions, mechanisms, and identification. Theranostics. (2020) 10:3503–17. doi: 10.7150/thno.42174, PMID: 32206104 PMC7069073

[174] Wawrzyniak O Zarębska Ż Kuczyński K Gotz-Więckowska A Rolle K . Protein-related circular RNAs in human pathologies. Cells. (2020) 9. doi: 10.3390/cells9081841, PMID: 32781555 PMC7463956

[175] Dlamini Z Ladomery MR Kahraman A . Editorial: The RNA revolution and cancer. Front Endocrinol. (2024) 15:1422599. doi: 10.3389/fendo.2024.1422599, PMID: 38832352 PMC11144892

[176] Ferrero G Licheri N De Bortoli M Calogero RA Beccuti M Cordero F . Computational analysis of circRNA expression data. Methods Mol Biol (Clifton NJ). (2021) 2284:181–92. doi: 10.1007/978-1-0716-1307-8_10, PMID: 33835443

[177] Dong J Zeng Z Sun R Zhang X Cheng Z Chen C . Specific and sensitive detection of CircRNA based on netlike hybridization chain reaction. Biosensors bioelectronics. (2021) 192:113508. doi: 10.1016/j.bios.2021.113508, PMID: 34284304

[178] Mi Z Zhongqiang C Caiyun J Yanan L Jianhua W Liang L . Circular RNA detection methods: A minireview. Talanta. (2022) 238:123066. doi: 10.1016/j.talanta.2021.123066, PMID: 34808570

[179] Zhang P Gao K Liang Y Su F Wang F Li Z . Ultrasensitive detection of circular RNA by accurate recognition of the specific junction site using stem-loop primer induced double exponential amplification. Talanta. (2020) 217:121021. doi: 10.1016/j.talanta.2020.121021, PMID: 32498896

[180] Huang D Zhu X Ye S Zhang J Liao J Zhang N . Tumour circular RNAs elicit anti-tumour immunity by encoding cryptic peptides. Nature. (2024) 625:593–602. doi: 10.1038/s41586-023-06834-7, PMID: 38093017

[181] Li W Liu JQ Chen M Xu J Zhu D . Circular RNA in cancer development and immune regulation. J Cell Mol Med. (2022) 26:1785–98. doi: 10.1111/jcmm.16102, PMID: 33277969 PMC8918416

[182] Yang L Fu J Zhou Y . Circular RNAs and their emerging roles in immune regulation. Front Immunol. (2018) 9:2977. doi: 10.3389/fimmu.2018.02977, PMID: 30619334 PMC6305292

[183] Ying K Chen J Fu Z Ren B . FAS-mediated circRNA-miRNA-mRNA crosstalk network regulates immune cell infiltration in cerebral infarction. J Mol neuroscience: MN. (2023) 73:117–28. doi: 10.1007/s12031-023-02100-7, PMID: 36656441

[184] Zhou Z Sun B Huang S Zhao L . Roles of circular RNAs in immune regulation and autoimmune diseases. Cell Death Dis. (2019) 10:503. doi: 10.1038/s41419-019-1744-5, PMID: 31243263 PMC6594938

[185] Du A Yang Q Sun X Zhao Q . Exosomal circRNA-001264 promotes AML immunosuppression through induction of M2-like macrophages and PD-L1 overexpression. Int Immunopharmacol. (2023) 124:110868. doi: 10.1016/j.intimp.2023.110868, PMID: 37657244

[186] Guan H Tian K Luo W Li M . m(6)A-modified circRNA MYO1C participates in the tumor immune surveillance of pancreatic ductal adenocarcinoma through m(6)A/PD-L1 manner. Cell Death Dis. (2023) 14:120. doi: 10.1038/s41419-023-05570-0, PMID: 36781839 PMC9925427

[187] Han R Rao X Zhou H Lu L . Synergistic immunoregulation: harnessing circRNAs and piRNAs to amplify PD-1/PD-L1 inhibition therapy. Int J nanomedicine. (2024) 19:4803–34. doi: 10.2147/IJN.S461289, PMID: 38828205 PMC11144010

[188] Liang L Gao M Li W Tang J He Q Zeng F . CircGSK3β mediates PD-L1 transcription through miR-338-3p/PRMT5/H3K4me3 to promote breast cancer cell immune evasion and tumor progression. Cell Death Discov. (2024) 10:426. doi: 10.1038/s41420-024-02197-8, PMID: 39366935 PMC11452702

[189] Ye Z Ding J Huang J Hu Z Jin F Wu K . Ginsenoside Rg3 activates the immune function of CD8+ T cells via circFOXP1-miR-4477a-PD-L1 axis to induce ferroptosis in gallbladder cancer. Arch pharmacal Res. (2024) 47:793–811. doi: 10.1007/s12272-024-01516-y, PMID: 39466543

[190] Zhang M Wan L Zhang X Wang S Li F Yan D . Exosome circ-CBLB promotes M1 macrophage polarization in rheumatoid arthritis through the TLR3/TRAF3 signaling axis. Front Immunol. (2025) 16:1627389. doi: 10.3389/fimmu.2025.1627389, PMID: 40746530 PMC12310475

[191] Zhang Z Zhang X Zhang Y Li J Xing Z Zhang Y . Spinal circRNA-9119 Suppresses Nociception by Mediating the miR-26a-TLR3 Axis in a Bone Cancer Pain Mouse Model. J Mol neuroscience: MN. (2020) 70:9–18. doi: 10.1007/s12031-019-01378-w, PMID: 31368062

[192] Zhang Z Zhang X Zhang Y Li J Xing Z Zhang Y . Retraction Note to: Spinal circRNA-9119 Suppresses Nociception by Mediating the miR-26a-TLR3 Axis in a Bone Cancer Pain Mouse Model. J Mol neuroscience: MN. (2020) 70:1926. doi: 10.1007/s12031-020-01693-7, PMID: 32875539

[193] Zhang Z Zhang X Zhang Y Li J Xing Z Zhang Y . Correction to: Spinal circRNA-9119 Suppresses Nociception by Mediating the miR-26a-TLR3 Axis in a Bone Cancer Pain Mouse Model. J Mol neuroscience: MN. (2020) 70:19–20. doi: 10.1007/s12031-019-01407-8, PMID: 31713153

[194] Ghazimoradi MH Babashah S . The role of CircRNA/miRNA/mRNA axis in breast cancer drug resistance. Front Oncol. (2022) 12:966083. doi: 10.3389/fonc.2022.966083, PMID: 36132137 PMC9484461

[195] Li H Wang C Jin Y Cai Y Sun H Liu M . The integrative analysis of competitive endogenous RNA regulatory networks in osteoporosis. Sci Rep. (2022) 12:9549. doi: 10.1038/s41598-022-13791-0, PMID: 35680981 PMC9184474

[196] Li M Duan L Li Y Liu B . Long noncoding RNA/circular noncoding RNA-miRNA-mRNA axes in cardiovascular diseases. Life Sci. (2019) 233:116440. doi: 10.1016/j.lfs.2019.04.066, PMID: 31047893

[197] Rong D Sun H Li Z Liu S Dong C Fu K . An emerging function of circRNA-miRNAs-mRNA axis in human diseases. Oncotarget. (2017) 8:73271–81. doi: 10.18632/oncotarget.19154, PMID: 29069868 PMC5641211

[198] Su K Cui X Zhou J Yi Q Liu O . Construction of an interactome network among circRNA-miRNA-mRNA reveals new biomarkers in hBMSCs osteogenic differentiation. Sci Rep. (2024) 14:24507. doi: 10.1038/s41598-024-76136-z, PMID: 39424659 PMC11489463

[199] Wang R Li Q Chu X Li N Liang H He F . Sequencing and Bioinformatics analysis of lncRNA/circRNA-miRNA-mRNA in Glioblastoma multiforme. Metab Brain Dis. (2023) 38:2289–300. doi: 10.1007/s11011-023-01256-w, PMID: 37389689

[200] Fu G Qiu L Wang J Li S Tian J Wu J . Genome-wide characterization of circular RNAs in three rat models of pulmonary hypertension reveals distinct pathological patterns. BMC Genomics. (2025) 26:127. doi: 10.1186/s12864-025-11239-z, PMID: 39930385 PMC11812181

[201] Hashemi M Khoushab S Aghmiuni MH Anaraki SN Alimohammadi M Taheriazam A . Non-coding RNAs in oral cancer: Emerging biomarkers and therapeutic frontier. Heliyon. (2024) 10:e40096. doi: 10.1016/j.heliyon.2024.e40096, PMID: 39583806 PMC11582460

[202] Zhou Z Zhang J Zheng X Pan Z Zhao F Gao Y . CIRI-deep enables single-cell and spatial transcriptomic analysis of circular RNAs with deep learning. Advanced Sci (Weinheim Baden-Wurttemberg Germany). (2024) 11:e2308115. doi: 10.1002/advs.202308115, PMID: 38308181 PMC11005702

